# Aging with Disability Symptoms and Ability to Participate in, and Satisfaction with, Social Participation among Persons Aged 45–64

**DOI:** 10.3390/healthcare10050903

**Published:** 2022-05-13

**Authors:** Michelle Putnam, Kerri Morgan, Rachel Heeb, Yan Yan, Szu-Wei Chen, Susan L. Stark

**Affiliations:** 1 School of Social Work, Simmons University, 300 The Fenway, Boston, MA 01602, USA; 2 Department of Occupational Therapy, Washington University School of Medicine in St. Louis, 4444 Forest Park Avenue, St. Louis, MO 63108, USA; morgank@wustl.edu (K.M.); heebr@wustl.edu (R.H.); szu-wei.chen@wustl.edu (S.-W.C.); starks@wusm.wustl.edu (S.L.S.); 3 Department of Surgery, Washington University School of Medicine in St. Louis, 4444 Forest Park Avenue, St. Louis, MO 63108, USA; yany@wustl.edu

**Keywords:** aging, disability, social participation

## Abstract

Pain, fatigue, and depression, considered aging with disability (AwD) symptoms, are known to be substantially higher among middle-aged adults with long-term disability compared to their age peers. Participation has been recognized as an important component of health. This cohort survey study reports findings on the relationship between AwD symptoms and ability to participate in, and satisfaction with participation in, social roles and activities using PROMIS measures. Data were collected at three time points from individuals aged 45–64 with an average of two decades of disability duration and primarily living in the state of Missouri, USA. This study reports on Time 1 (T1) and Time 3 (T3), pre- and post-COVID-19 pandemic declaration, respectively. Multiple regressions using both individual AwD symptoms and a composite measure demonstrated that having more pain, fatigue, and depression was associated with worse participation outcomes. Lower physical function scores were also related to lower participation scores, as was being female and living with others, and having more income reduced participation. Better physical health and identifying as African American/Black were associated with higher participation scores. Our findings suggest that AwD symptoms, along with other sociodemographic and health factors, play a substantial role in the social participation outcomes for persons aging with disability and remain consistent over time.

## 1. Introduction

### 1.1. Social Participation as a Component of Health for Persons Aging with Long-Term Disabilities

Calls for including social participation for older adults and persons with disabilities within a broad conceptualization of individual health and wellness have been made globally. The World Health Organization describes healthy aging as “creating the environments and opportunities that enable people to be and do what they value throughout their lives”, regardless of a person’s health or functional status [1], and its Global Network for Age-Friendly Cities and Communities emphasizes full participation of older people to promote healthy and active aging [2]. These priorities are also aligned with the United Nations Declaration of Rights for Persons with Disabilities, which, in Article 3(c), calls for “full and effective participation and inclusion in society” [3]. At the national level, governments are advancing various initiatives on disability and inclusion. For example, in the United States (U.S.), explicit goals within the Centers for Disease Control and Prevention’s Healthy People 2030 initiative include increasing the accessibility of housing [4] and employment among people with disabilities [5], as well as the number of states and territories that have specific health promotion programs for persons with disabilities [6]. The United Kingdom’s National Disability Strategy, launched in 2021, has an even more extensive set of aims across all government sectors to foster inclusion and participation [7].

These calls are supported by an established and growing international body of evidence demonstrating the relationship between participation and mental and physical health among older adults [8] and persons with disabilities [9]. Systematic reviews have consistently identified personal demographic factors, social support, attitudes of others, physical environment, technology, and services and policies as having the potential to act as both barriers and facilitators to participation among adults with disabilities of all ages [10,11]. Studies investigating social inclusion interventions for older adults and people with disabilities exist, but calls to improve this evidence base have been made [12]. Systematic reviews have found that interventions to improve participation and inclusion are substantially varied in their focus and that there is limited evidence of long-term effectiveness, including for technological interventions [13], occupational therapy interventions [14], and environmental and psychosocial interventions connected to age-friendly communities [15].

Thus, it is clear that there is an international public health and civic desire to support the engagement, participation, and inclusion of older adults and people with disabilities; however, advancing an evidence-based agenda to support these aims will require continued research. We also believe that to advance knowledge in this field, more nuanced consideration should be given to subpopulations where the intersection of health, disability, and aging may create unique physiological symptoms that influence participation and social participation in particular. Specifically, we recognize the need to better understand participation among people who have lived with disability for many years, as they may experience prolonged social exclusion and barriers to participation.

Our specific focus is on individuals in midlife who have lived with disability for an extended period of time. This subgroup ranges from individuals who have lived with disability since birth to those who acquired disability in early or midlife. In the research literature, these individuals are often termed “persons aging with disability” and tend to report common symptoms, including pain, fatigue, and depression, at higher rates than the general population, although levels of these symptoms often vary [16,17]. Whereas persons of any age can grow older with disability and may have shared predictors of participation, including health [18], functional independence, and income [19], midlife is a period where individuals aging with disability (AwD) may begin to reduce participation in areas such as work, social engagement, or other activities because of increasing difficulty in doing them. In disability, rehabilitation, and aging literature, there is emerging evidence that pain, fatigue, and depression consistently influence the daily living experiences of persons aging with disability. We want to better understand how these symptoms influence social participation. However, some of the most compelling data available to inform interventions are more than a decade old, and much of the research completed in the U.S. has been conducted with samples predominantly identifying as White race/ethnicity. Additionally, all of the research we have found reports on studies undertaken prior to the COVID-19 pandemic, which has had profound implications for persons with disabilities.

Our cohort study of persons aging with disability, as reported elsewhere [20], has the aim of understanding participation for persons with long-term physical disabilities aged 45–64. It is anchored in a community-based research network (CBRN) composed of regional aging and disability organizations. A larger goal and commitment of our center (CEDARMidwest.org) is to help the CBRN and other aging and disability organizations identify instruments and tools to efficiently collect meaningful data, leverage their existing resources, and identify interventions that can help them support, sustain, and/or restore individual participation in important life activities [21].

In this paper, we explore demographic and disability correlates and predictors of changes in social participation among persons with physical disabilities aged 45–64 using two data waves: one prior to the onset of the COVID-19 pandemic and one post-onset. Our aim is to help build usable knowledge for this aging subgroup of persons with disabilities in order to support participation and inclusion.

### 1.2. Social Participation

Participation has been articulated by the World Health Organization, through the International Classification of Functioning, Disability and Health (ICF), as a broad construct—a person’s involvement in all areas of life—which includes social engagement in life activities [22]. Some researchers suggest that social participation is a more focused domain. For example, Douglas, Georgiou, and Westbrook [23] identified only three domains of social participation—social connections, informal social participation, and volunteering—in their review of the relationship between social participation and health outcomes for older adults. Others take a more global perspective. Smallfield and Molitor’s [24] systematic review of occupational interventions supporting social participation and leisure engagement among older adults (2018) included several dozen specific terms related to the themes of social participation and leisure in their literature search. They then classified interventions under equally broad categories. Engel-Yeger et al. [25] completed a scoping review of participation outcomes following stroke and found 34% of the articles they evaluated used the ICF framework, but they identified 22 different measures of participation used.

In an effort to advance the practical evaluation of participation in a way that is meaningful to persons with disabilities, Hammel and colleagues [26], in a qualitative investigation, asked people with disabilities with a mix of diagnoses, impairments, and conditions to operationalize the term “participation”, and they identified social participation—expressed as being a part of an activity, group, social situation, context, or relationship—as a core component of the concept. Later, Hammel collaborated with colleagues to identify barriers and facilitators to participation, including social participation; these ranged from elements of the built environment to social supports and economic resources [27]. Building on that and other work, Martin Ginis et al. [28] completed a systematic review of definitions and conceptualizations of subjective perceptions of participation among disability populations in an effort to identify experiential aspects of the concept, identifying six elements to be considered in its operationalization. This is just a small portion of the relevant research related to conceptualizing and measuring participation.

In our study, we used the PROMIS (Patient-Reported Outcomes Measurement Information System) measures of “ability to participate in social roles and activities” and “satisfaction with ability to participate in social roles and activities” [29,30,31]. Recent reviews have noted that PROMIS participation measures may not capture all elements of the ICF framework [32,33]; however, they have been employed to explore social participation among persons aging with disability in single and cross-disability samples.

### 1.3. Pain, Fatigue, and Depression

Pain, fatigue, and depression are commonly measured and regularly reported to be significant indicators of health, wellness, and participation among persons who have lived long-term with disability. Depending on the study, these symptoms are not always individually significant in their association with participation, but they commonly are. For example, Salter et al. [34] examined the role of fatigue and the PROMIS measure “ability to participate in social roles and activities” in a large sample of individuals with multiple sclerosis (MS) and found both pain and depression, as well as severity of disability, significantly predicted levels of fatigue. The researchers also found that as fatigue increased, ability to participate decreased. Their study employed both a PROMIS fatigue measure and the Fatigue Performance Scale; both measures produced similar results. Murphy et al. [35] reported similar findings in a fatigue management intervention study of persons with systemic sclerosis with a much smaller sample size. In that study, higher levels of fatigue and worse physical function were significantly associated with lower levels of ability to participate in social roles and activities; self-efficacy education did not moderate this relationship post-intervention. Pokryszko-Dragan et al. [36] did not use PROMIS measures but, rather, a different set of standard measures, including the World Health Organization Disability Assessment Schedule 2.0, to assess social functioning. They also found social participation to be significantly correlated with depression, fatigue, and mobility problems among a Polish sample of persons with MS. In contrast to these findings, however, in a large cross-diagnostic sample, Hreha et al. [37] found that pain and fatigue did not significantly predict ability to participate in social roles using the PROMIS measure, but depression did. In all four of these studies, the samples were predominantly female, with an average duration of disability of more than 10 years.

Studies with predominantly male participants report similar findings. Lundström et al. [38] assessed participation among persons aging with traumatic spinal cord injury using 10 domains of the PARTS/M-v3 (PARTicipation Survey/Mobility version-3), of which most fall into the operationalization of social participation described by Hammel et al. [26]. The largely male sample had an average age of 62 and mean time since injury of 35.5 years. Study findings reported that pain, fatigue, and a grouped set of secondary health conditions (including depression) influenced participation in each activity domain. Open-ended participant responses identified an expected increased need for support to participate in activities within the next five years. Kuzu et al. [39] reported similar findings using the PROMIS ability to participate measure and the PROMIS measures of pain, fatigue, depression, and anxiety among a predominantly male sample of persons with spinal cord injury. Their study employed 7-day diaries to track symptoms and participation. Findings indicated that in general, individuals with more pain and fatigue participate less. However, on a daily basis, less fatigue and depression were associated with greater participation, whereas pain intensity and anxiety had no relationship with participation.

There is some indication that pain, fatigue, and depression significantly influence participation among adults, regardless of age of disability onset, and that changes in these symptoms over time may reduce participation. Hilberink et al. [40] explored variance in participation among adults with disabilities in the Netherlands aged 40 and older, grouping their sample into early and late disability-onset groups to consider self-reports of changes in symptoms and participation over time. They found that persons with onset of disability prior to age 25, as well as those with onset after age 25, both experienced high levels of pain, fatigue, and depression. However, individuals with early disability onset reported worsened pain and fatigue after age 40 than those with later disability onset. That said, more than 70% of participants in both groups reported considerable worsening of walking ability and energy levels, and a majority reported worsening pain and fatigue after age 40. About 40% of each group reported an increase in the regularity of feelings of depression. Sample members in both groups reported participation declines in self-care and social relationships, as well as engagement in fewer activities over time. Battalio et al. [41] also undertook an analysis of participation over time, using data from the University of Washington’s longitudinal survey of adults aging with disability, which includes persons with MS, postpoliomyelitis syndrome, and muscular dystrophy. Using the PROMIS measure of satisfaction with ability to participate in social roles and activities, Battalio et al. evaluated change over approximately three years. Their analysis found that physical function and secondary health conditions, including pain and fatigue, as well as chronic medical comorbidities, accounted for more than half of the variance in role satisfaction at Time 1 but only 3% of the variance in change in social role satisfaction at Time 2. Only mood and energy (i.e., depression and fatigue) demonstrated a significant relationship with change in satisfaction. Thus, there is some consistency in findings that pain, fatigue, and depression do influence social participation, but there is a need for additional research to increase the depth of evidence in this area.

The analysis we report here adds to the evidence base concerning the associations of pain, fatigue, and depression with satisfaction with social roles and the ability to participate in social roles for persons aging with disability. Specifically, we evaluated two different data waves from our cohort study composed of a diverse community-based sample. We compared the use of pain, fatigue, and depression as independent factors against a composite measure of AwD symptoms (pain, fatigue, and depression) to consider its use as a latent measure, given the commonality with which all three symptoms are regularly found to be significant predictors of participation among persons aging with disability.

## 2. Materials and Methods

### 2.1. Participants and Procedures

Our data are from a longitudinal cohort study, collecting survey data once a year for 3 years at 12-month intervals: study enrollment (T1), collected August 2018–July 2019; 1-year follow-up (T2), collected August 2019–July 2020; and 2-year follow-up (T3), collected August 2020–August 2021. In this study, we analyzed data from T1 and T3 only. The WHO declared COVID-19 a pandemic on 11 March 2020, [42] m approximately halfway through data collection for T2. Pandemic-related closures, social distancing restrictions, and other social engagement guidelines implemented after March 11 were in place for many months, thereby artificially modifying social participation of our sample members during T2 data collection. For this reason, we did not evaluate data longitudinally across data waves. We also did not evaluate change in social participation from T1 to T3 in this paper, as the attrition at Wave 3 would reduce the total number of participants available for analysis at T1. Here, we report only T1 and T3 data to assess models of factors influencing social participation and their consistency at different time points. We intend to review Wave 2 data at a later time and consider how the pandemic onset may have affected differences in participant responses during that year’s data collection.

Ethics approval was granted by Washington University School of Medicine in St. Louis. Cohort baseline (T1) demographics and methodology have been previously reported [20].

Participant inclusion criteria were: aged 45–65 years, duration of self-reported disability of 5 years or more at the time of recruitment, English-speaking, and ability to autonomously provide consent. Participants both provided consent and completed the survey online or over the phone. The average time for completion was 45–60 min for all three time points. Accessibility-related assistance was provided upon request. We assumed a 25% attrition rate over time, calculating a need for 470–500 participants for sufficient statistical power. Using a range of in-person and online recruiting techniques resulted in 474 unique participant responses at T1, 386 participant responses at T2, and 326 participant responses at T3 (69% response rate). Three participants answered the T3 survey but did not participate in all 3 data waves; specifically, 2 participants only completed T3, and 1 participant completed T1 and T3 only. We do not analyze individuals longitudinally in this analysis but considered T1 and T3 data separately, so we left those individuals in the T3 data.

### 2.2. Measures

The assessments for all three time points consist of self-reports of health, disability, and social support characteristics; activity, participation, and environmental factors; and long-term service and support use. Measures were selected in consultation with the CBRN [21]. All survey questions were identical for online and telephone administrations. Trained graduate assistants administered the phone survey while completing data entry using REDCap (Research Electronic Data Capture), a secure, web-based application [43]. The online survey was sent out directly via REDCap. In this paper, we report the sociodemographic, health, disability, and social participation measures at T1 and T3 of the longitudinal cohort survey. Sociodemographic, self-rated health, physical function, and aging with disability symptoms served as independent variables. Social participation measures served as dependent variables.

Sociodemographic measures included age, race and ethnicity, gender, mean years living with self-reported disability, marital status, education, living arrangement, employment status, food security, and annual personal income (categorized as ≤USD10,008 or >USD 10,008, the state of Missouri’s income ceiling in 2018 for qualification for Medicaid, which is one type of public health insurance in the U.S., or above this income level).

Self-rated physical and mental health were both measured on a five-point scale (1 = excellent, 5 = poor). We also employed four different measures from the PROMIS^©^ [44], including (1) PROMIS Physical Function with Mobility Aid Short Form [44,45], which has been validated with persons with physical disabilities and measures ability to perform daily living activities [46]; (2) PROMIS Fatigue Profile; (3) PROMIS Pain Interference measure; and (4) PROMIS Depression measure [46]. The latter three measures evaluate presence and intensity of conditions commonly reported as AwD symptoms and were measured with PROMIS computerized adaptive testing (CAT) versions. These three measures use a five-point scale, with higher scores representing higher levels of the symptom over 7 days. T scores generated from all PROMIS scales were compared against the general American adult population with a mean score of 50 and standard deviation of 10. Scores greater than 50 on the physical function PROMIS measure were interpreted to represent better physical function, whereas scores lower than 50 were interpreted to represent worse physical function. Scores higher than 50 for pain, fatigue, and depression represent feeling worse than the average adult, whereas scores lower than 50 represent feeling better than the average adult does. We used the Washington Group Short Set on Functioning to collect data on type of functional disability [47]. Participants were also asked to self-report their primary cause of disability in an open-ended question; responses were qualitatively categorized using the federal U.S. Social Security Disability Insurance program medical specialty codes as a general reference for grouping reported conditions [48].

We measured social participation using (1) the PROMIS Adult Ability to Participate in Social Roles and Activities-CAT version, which is not time-bound and assesses the perceived ability to perform one’s usual social roles and activities [30] and (2) the PROMIS Satisfaction with Social Roles and Activities-CAT version [31], which assesses self-reported contentment with social roles, such as work and family responsibilities, in the past 7 days. Each uses a five-point Likert scale. Satisfaction with Social Roles and Activities is scored: not at all = 1, very much = 5. Ability to Participate in Social Roles and Activities is scored: never = 5, always = 1; the scale was reversed for analysis. Satisfaction with Social Roles and Activities has 44 items, although the CAT requires only a minimum of four items be answered to produce a score. Ability to Participate in Social Roles and Activities has five items, with the same CAT scoring requirement. As with the prior PROMIS measures, they were compared to the American adult population with a mean score of 50 and standard deviation of 10. Higher scores represent better abilities and more satisfaction. All six of the PROMIS measures are included in the Supplementary file available online.

### 2.3. Statistical Analysis

We used SAS/STAT software (version 9.4, SAS Inc., Cary, NC, USA) [47] for analysis, setting significance at *p* ≤ 0.05. We explored attrition by performing a series of chi-square tests to compare the differences in year 1 sociodemographic, health traits, and AwD symptoms between participants who participated in the year 3 survey versus those who did not. The same groups of comparison were also performed using independent t test to examine the differences in all the year 1 PROMIS measures and years of having primary disability. We then explored differences in two social participation outcomes across different sociodemographic groups and health trait levels by using two-sample t tests (two levels of categorical variables) or ANOVA (more than two levels of categorical variables). In prior analysis, we reported these results for T1 [20].

Following that, for T1 and T3, we conducted a series of correlation analyses to explore associations between measures, including a selected set of continuous sociodemographic, health, and AwD symptom measures. Based on the bivariate and correlation analyses, we selected a set of variables to include in a multivariable regression model that we ran for both T1 and T3 in parallel. Our aim was to consider T1 and T3 data separately in order to determine whether the same variables were significant at each time point. Because we were uncertain as to how the COVID-19 pandemic would affect the data, we did not evaluate change in social participation scores for individuals but, rather, aimed to understand whether the predictive model for T1 would be the same as that for T3.

As in other studies, we entered pain, fatigue, and depression into our multiple linear regression model as three independent variables and evaluated those results. Then, in order to preserve more information and avoid multicollinearity problems, we decided to use a factor analysis to obtain the factor score for these three commonly reported AwD symptoms [16,46,49,50] of fatigue, pain, and depression and repeat the regression analysis. Doing so was based on our knowledge, as we assumed there was an underlying concept/latent variable measured by these three commonly reported symptoms of AwD. We used factor analysis to obtain scores to rank our participants along the underlying construct. The latent factor variable was assumed to have standard normal distribution, with about 99% of values in the range between −3 and 3, a mean of 0, and a standard deviation of 1. Higher scores represent worse AwD symptoms. We used the first factor score from the factor model as one independent variable in the model. The first factor score explained 69.96% of variation from fatigue, pain, and depression, and the factor loadings on these three variables were substantial, with all of them above 0.8 (depression, 0.82; fatigue, 0.85; and pain, 0.83). Because there was only one factor, we did not use any rotation. These results provided supporting evidence for our assumption of a latent variable measured by fatigue, pain, and depression.

## 3. Results

The sample demographics were similar between T1 and T3 despite attrition. Two-thirds of participants were female, most were single, less than a quarter were working, and the majority lived alone. For T1, the mean age was 56.8 years (SD = 5.6), and at T3, the mean age was 55.5 years (SD = 5.7). T1 participants had a mean of 19.0 years living with their disability (SD = 13.7, range = 5–65 years); this increased to 20.9 at T3 (SD = 13.63, range = 7–65 years). Participants were asked their primary cause of disability at T1, but this question was not repeated at T3. At T1, 37% of participants reported disability related to neurological conditions (e.g., cerebral palsy, multiple sclerosis, spinal cord disorders, traumatic brain injury), and 26% reported musculoskeletal conditions (e.g., degenerative and osteoarthritis, spinal stenosis, amputation). At T1, 94% of participants reported functional difficulty (any difficult vs. no difficulty) with walking and climbing steps, 63% with remembering and concentrating, 53% with seeing, 52% with self-care, 25% with hearing, and 22% with communicating. T3 participants reported similar rates of any functional difficulty: 88% walking and climbing steps, 62% remembering and concentrating, 51% with seeing, 46% with self-care, 27% with hearing, and 22% with communicating. At T1, 34% of participants reported one to two functional difficulties, 48% reported three to four, and 17% reported five to six functional difficulties. At T3, 35% of participants reported one to two functional difficulties, 47% reported three to four, and 14% reported five to six functional difficulties.

### 3.1. Sociodemographic, Health, and AwD Symptom Traits in T1 and T3 Samples

Table 1 displays T3 sample traits and, based on chi-square tests, indicates which sample traits were significantly different at T1 and T3 due to cohort attrition. In the T3 sample (vs. T1 sample), a slightly higher proportion of participants identified as White (62% vs. 61%) and Black/African American (28% vs. 26%), and fewer identified as another race/ethnicity (10% vs. 12%). The T3 sample also skewed slightly higher in terms of education attainment, with a greater proportion of participants having a bachelor’s or graduate degree at T3 than T1 (39% vs. 33%), a lower proportion having an associate degree (equivalent to 2 years of university training) or some college/training (35% at T3 vs. 38% at T1), and only 26% holding a high-school diploma or less at T3 compared to 29% at T1. A higher proportion of individuals had personal income of more than USD 10,008 per year at T3 (78% vs. 65%). Finally, participants at T3 self-reported being in better health than those at T1 (31% vs. 28% reported excellent/very good health, 35% vs. 32% in good health, 38% vs. 41% in fair health, and 15% vs. 24% in poor health). Thus, the most notable differences between participants in the T1 sample and those in the T3 sample were race/ethnicity, education, income, and self-reported physical health.

Participants’ average physical function T score was higher and AwD symptoms were lesser at T3 than at T1. T1 scores are reported elsewhere [20] (Cite F1000 paper). Mean T scores and standard deviations (SD) at T3 were: physical function, 36.51 (SD = 8.91); fatigue, 56.46 (SD = 10.40); pain, 58.33 (SD = 10.34); and depression, 52.67 (SD = 9.86). The mean T score for ability to participate in social roles and activities at T3 was 45.53 (SD = 9.29), and for satisfaction with social roles and activities, the mean T score was 44.26 (SD = 9.50). Thus, similarly to participants at T1, participants at T3 had a lower mean average ability to participate in and satisfaction with social roles and activities.

At T1, independent T tests and ANOVAs found significant between group differences in T scores of ability to participate in social roles and activities between/among people of different personal income, age group (45–54, 55–60, 61–65), race/ethnicity, and educational attainment. Participants with different living arrangements, food security, employment status, age group, race/ethnicity, and educational attainment had significant differences in T scores for satisfaction with social roles and activities. At T3, independent t tests and ANOVAs found significant differences in T scores for ability to participate in social roles and activities between people of different genders and with different experiences of food security. Food security and employment status presented significant group differences in T scores for satisfaction with social roles and activities.

### 3.2. Correlations between Selected Traits and Social Participation Outcomes at T1 and T3

Table 2 presents correlations between selected variables and both PROMIS social participation measures for T1 and T3. Correlations for the AwD symptoms composite variable were significant, as were individual correlations with pain, fatigue, and depression. Based on the univariate and bivariate analyses and our prior knowledge, we selected measures to include in the regression models predicting ability to participate in social roles and activities (Table 3) and satisfaction with social roles and activities (Table 4).

### 3.3. Multivariate Regressions for Social Participation Outcomes at T1 and T3

We ran parallel regression models for two social participation measures; each model contained either the AwD symptoms composite score or individual fatigue, pain, and depression variables. The parallel models were applied using both T1 and T3 data. A total of eight regression models were applied (Table 3 and Table 4). Table 3 displays models for ability to participate in social roles and activities, showing that under both T1 and T2, the R-square statistics are nearly identical between the model including the AwD composite score (T1 *R^2^* = 0.435, T3 *R^2^* = 0.486) and the model including the three individual symptom variables (pain, fatigue, and depression) (T1 *R^2^* = 0.436, T3 *R^2^* = 0.486). We report White robust standard errors (SE), as they mainly deal with heterogeneity in the residual variance, which some authors [51] claim also alleviates the impact of non-normality, outlier, and influential observations on statistical inference.

The multiple regression results in Table 3 indicate that at T1 and T3, experiencing worse pain, fatigue, and depression was associated with reduced ability to participate. When we included the AwD symptoms composite score, AwD symptoms accounted for the largest amount of variance (T1 *b* = −4.61, *t* = −10.78, *p* < 0.001; T3 *b* = −5.08, *t* = −9.41, *p* < 0.001) in this outcome. Better physical function was consistently associated with greater ability to participate at T1 and T3. Additionally, higher personal income was associated with less ability to participate at T1 and T3. Identifying as Black/African American, as opposed to White, was associated with better ability to participate at T1 but not at T3. Older age and being female were associated with reduced ability to participate at T3 but not at T1.

Table 4 displays models for satisfaction with social roles and activities. It shows the same patterns with R-square and b-coefficient significance of AwD symptoms (composite and individual variables) across models at T1 and T3, with one exception. Specifically, at T3, the AwD symptoms composite score is significant, and so are fatigue and depression in the other model, but not pain (*b* = −0.06, *t* = −1.1, *p* > 0.05). Thus, we concluded that whereas the composite measure is parsimonious and may aid data interpretation, it is also important to individually evaluate the three separate measures of pain, fatigue, and depression.

In Table 4, multiple regression results for satisfaction with social roles and activities were similar to results for ability to participate, with a few notable differences. At T1 and T3, individuals with more AwD symptoms and higher incomes were associated with lower levels of satisfaction. Notably, at T1, living with others was also associated with lower satisfaction, and being Black/African American was associated with higher satisfaction. However, at T3, neither of these two variables was significantly associated with satisfaction, nor was pain. Instead, having longer years living with disability and better physical function were associated with higher levels of satisfaction.

## 4. Discussion

Our analysis indicates that AwD symptoms—pain, fatigue, and depression—are consistently associated with the ability to participate in, and satisfaction with, social roles and activities at T1 and T3. In sum, our findings suggest that individuals who experience greater levels of pain, fatigue, and depression may have less ability to participate in, and less satisfaction with their participation in, social roles and activities. Overall, our findings, when placed in the context of prior research, add to the growing evidence that AwD symptoms contribute to variance in social participation outcomes for middle-aged adults living with long-term disability. This is important, as these symptoms have potential to be managed and reduced through therapeutic medical and non-medical interventions.

We also found that lesser physical function was also associated with worse social participation outcomes. Here, health and social interventions may play a positive role. Clarke et al. [52] found pain, fatigue, physical function, environmental factors, and perceived social support all significantly influenced ability to participate, using the same PROMIS ability to participate measure as we did. Additional exploration of the relationship between AwD symptoms, physical function, and environmental factors is warranted. Expanding this knowledge base could help us understand how to support inclusion and participation as individuals with disabilities grow older.

Additionally, our findings suggest that personal income, self-identified race, living arrangement, and gender may influence social participation. Across all eight regression models, being financially better-off was associated with less-than-average participation scores. This was an unexpected finding.

Research on the intersection of income, participation, and aging with disability is quite modest; however, evidence presented by Shuey and Wilson [53] from a longitudinal analysis of the Panel Study of Income Dynamics suggest that onset of disability at any age leads to substantially increased risk of poverty over time. Better understanding of ways income influences participation for persons AwD seems important for reducing barriers to inclusion. Within the context of the U.S. healthcare system, there is substantial variance in eligibility for the Medicaid insurance program for people who live in poverty depending on state. Medicaid provides more coverage of long-term service and supports for persons with disabilities than private insurance or Medicare insurance, which covers disabled workers. Having this insurance may promote greater social participation for individuals aging with disability, which can be seen in our previous findings [11]. Another explanation may be that adults with disability with higher incomes have higher expectations of participation, so those with more severe AwD symptoms may experience greater disappointment and, therefore, less satisfaction with their levels of participation. As such, a large percentage of our sample is no longer in the workforce but receives SSDI benefits, denoting prior workforce participation; this may influence our findings if these individuals would prefer to be engaged in paid work. We did not ask our respondents this question. The idea that higher income is associated with less satisfaction with social participation is inconsistent with evidence indicating that lower income is a barrier to participation among older adults and people with disabilities. [54] However, there may be limitations to our understanding of the relationship between these factors. Shandra [55], in her analysis of the American Time Use Survey data, explored four activity domains among adults aged 18–64 with and without disabilities and identified significant differences in time spent participating in market work, non-market work, and leisure activities. She found that age and social demographic traits (including income), as well as health factors, including self-rated health, use of pain medication, and feeling rested, were associated with differences in time spent participating in those activity domains. However, Shandra noted that as these measures accounted for between 28–50% of time use difference, further investigation was warranted to explain, as she termed it, the disability gap. Our findings suggest that there is much more to learn about the dynamic factors that influence participation among persons aging with long-term disability. This is an area we will further explore in our data and encourage others to as well.

In addition to the unexpected relationship with income, our finding that at T1, living with others was also associated with lower satisfaction with participation in social roles and activities was the opposite of what we anticipated. However, a similar finding was reported by Repke and Ipsen [56], who used the PROMIS measure of Satisfaction with Participation in Discretionary Social Activities to explore differences in social connectedness and perceived isolation among persons with disabilities in rural and urban areas. Their analysis identified general health and number of disability issues as being associated with satisfaction. Repke and Ipsen suggested that further understanding of the meaning of living alone for people with disabilities is warranted, as living alone may signify a level of functional or personal independence that living with others does not. They also cited Klinenberg’s [57] proposition that living alone is not equitable to loneliness and may encourage individuals to participate more with others in their daily lives. Given the age range of our study participants, these ideas are relevant to consider, as 58% lived with others at T1 but only 38% reported being married or partnered at that time. At T3, living situation was not associated with social participation satisfaction. This change at T3 may be related to the COVID pandemic guidelines, which encouraged social distancing, particularly for individuals with underlying health and chronic conditions.

In our analyses, we also found that racially identifying as an African American or Black individual was associated with more positive social participation outcomes. We were not able to identify any existing studies of persons aging with disability that had a similar finding. In the U.S., non-White individuals tend to have worse health and participation outcomes due, in part, to racial disparities in health equity in the U.S., for which there is overwhelming evidence [58,59]. There are some U.S.-based studies of participation among adults identifying as African American or Black with chronic disease but none that we identified with a comparative White sample. We recommend further exploration of the relationship between race and ethnicity, aging with disability, and social participation to gain additional knowledge to support and facilitate positive health outcomes.

Finally, at T3 but not at T1, being female reduced ability to participate in social roles and activities. Gender was not significant in terms of satisfaction with participation. We did not find satisfactory discussion of gender differences in participation in the aging with disability literature to explain this difference. This finding may be related to the COVID-19 pandemic in some way (e.g., restrictions imposed at the community level or other personal or community factors, changes in amount of time available for prior roles and activities); we are not sure. We recommend further exploration of gender differences in participation in future research and will further review other segments of our cohort data to better understand gender differences.

Based on our exploratory use of the composite measure of AwD symptoms, we believe there is potential for such a measure. In our regression analysis of ability to participate in social roles and activities, it performed equitably to the three independent variables of pain, fatigue, and depression. Arguably, it may be easier to interpret the effect of a composite measure than that of these three symptoms individually. However, the composite measure masked the non-significance of pain measure in our model of satisfaction with participation, so we recommend investigators run parallel analyses prior to making a determination to use the composite score.

## 5. Study Limitations

Limitations in our analysis include separate consideration of data from T1 and T3rather than longitudinal assessment of individual change. However, our aim was to better understand the role of AwD symptoms in modeling social participation. The few differences in demographics and health traits between T1 and T3 based on our attrition analysis permitted this assessment. Another limitation is that multiple regression analysis does not determine causality. Therefore, bidirectional relationships between the independent variables and social participation measures may be possible. Additionally, our T1 measure for personal income had only 2 levels, ≤USD 10,008 or >USD 10,009 which limits our ability to understand the relationship of income with social participation outcomes. Personal income at T2 and T3 was expanded into a multi- variable; we intend to further evaluate personal income at these time points to better understand the association. Finally, we were unable to determine whether differences between factors we found to be significant in predicting social participation outcomes at T1 and T3 are related to changes found among individual participants over time, the context of the COVID-19 pandemic, or something else. A strength of this analysis is the percentage of non-White participants in our sample (26% at T1 and 28% at T3).

## 6. Conclusions

Our findings expand the evidence that AwD symptoms are related to social participation outcomes. We recommend health and social care providers consider evaluating these symptoms when working with adults AwD in order to understand how they influence an individual’s social participation engagement. We recommend researchers, health and social care providers, and policy makers pay particular attention to participation outcomes among persons AwD in midlife, as reduced participation at this life stage may have implications for participation levels in later life, as well as health outcomes, as people grow older.

## Figures and Tables

**Table 1:** Sociodemographic traits of T3 participants with sample differences from T1 participants.

Sociodemographic Traits	T3 *n* = 326 *n* (%)	Significant Difference in Sample Distribution T1–T3
Chronological age, mean (SD)	55.5 yrs (SD = 5.7)	N/A
Years living with disability, mean (SD)	20.9 (SD = 13.63)	N/A
Sex at birth		-
Female	223 (69.04)	
Male	100 (30.96)	
Gender ^†^		-
Man	97 (30.41)	
Woman	222 (69.59)	
Race/ethnicity		X
White	202 (62.35)	
Black/African American	90 (27.78)	
Other	32 (9.88)	
Marital status		-
Currently married/partnered	117 (36.23)	
Single/widowed/other	206 (63.78)	
Educational attainment		X
High-school diploma or less	85 (26.23)	
Associate degree or some college/advanced training	114 (35.19)	
Bachelor’s degree/graduate degree	125 (38.58)	
Employment status		-
Paid work, full- or part-time	62 (19.31)	
Seeking paid work	11 (3.43)	
Retired, not seeking work, other	54 (16.82)	
Disability leave	194 (60.44)	
Living arrangement		-
Live alone	139 (43.17)	
Live with others	183 (56.83)	
Personal annual income		X
USD 10,008 or less	68 (21.73)	
USD 10,009 or more	245 (78.27)	
Self-rated physical health		X
Excellent/very good	49 (15.12)	
Good	105 (32.41)	
Fair	123 (37.96)	
Poor	47 (14.51)	
Self-rated mental health		-
Excellent/very good	102 (31.48)	
Good	112 (34.57)	
Fair	89 (27.47)	
Poor	21 (6.48)	

Notes: X = significant difference in within-variable categorical distribution at *p* ≤ 0.05, ^†^ The category of transgender was offered in the survey; however, no participants selected it.

**Table 2:** Correlations between selected variables and two social participation measures.

	Ability to Participate in Social Roles and Activities		Satisfaction with Social Roles and Activities	
Continuous Variables	Pearson’s r Correlation Coefficient		Pearson’s r Correlation Coefficient	
	T1	T3	T1	T3
Age (in years)	*r* = −0.03	*r* = 0.11 *	*r* = 0.05	*r* = −0.07
Depression	*r* = −0.51 ***	*r* = −0.51 ***	*r* = −0.51 ***	*r* = −0.49 ***
Physical function	*r* = 0.32 ***	*r* = 0.38 ***	*r* = 0.27 ***	*r* = 0.36 ***
Fatigue	*r* = −0.51 ***	*r* = −0.58 ***	*r* = −0.49 ***	*r* = −0.56 ***
Pain	*r* = −0.46 ***	*r* = −0.50 ***	*r* = −0.45 ***	*r* = −0.43 ***
AwD symptoms composite score ^†^	*r* = −0.59 ***	*r* = −0.63 ***	*r* = −0.58 ***	*r* = −0.58 ***
Years living with disability	*r* = 0.05 **	*r* = 0.11 *	*r* = 0.11 *	*r* = 0.15 **
	Spearman’s r Correlation coefficient		Spearman’s r Correlation coefficient	
Education	*r* = −0.04	*r* = 0.01	*r* = 0.05	*r* = −0.01
Self-reported physical health	*r* = 0.36 ***	*r* = −0.41 ***	*r* = 0.38 ***	*r* = −0.44 ***
Self-reported mental health	*r* = 0.33 ***	*r* = −0.38 ***	*r* = 0.34 ***	*r* = −0.39 ***

Note: * *p* < 0.05; ** *p* < 0.01; *** *p* < 0.001; ^†^ Composite measure includes pain, fatigue, and depression.

**Table 3:** Multivariate regression for ability to participate in social roles and activities at T1 and T3: AwD symptoms model comparisons.

	T1						T3					
	Composite AwD Symptoms			Individual AwD Symptoms			Composite AwD Symptoms			Individual AwD Symptoms		
Model Fit	*R^2^* 0.435 ***	*F*^(df1, df2)^ 28.72 ***^(12, 448)^		*R^2^* 0.436 ***	*F*^(df1, df2)^ 24.60 ***^(14, 446)^		*R^2^* 0.486 ***	*F*^(df1, df2)^ 22.92 ***^(12, 291)^		*R^2^* 0.486 ***	*F*^(df1, df2)^ 19.55 ***^(14, 289)^	
	*b*	*SE b*	*t*	*b*	*SE b*	*t*	*b*	*SE b*	*t*	*b*	*SE b*	*t*
Y intercept	42.90	4.10	10.47 ***	75.65	4.76	15.89 ***	49.80	4.48	11.11 ***	82.47	5.45	15.13 ***
Age (in years)	−0.01	0.06	−0.15	−0.01	0.06	−0.15	−0.17	0.07	−2.64 **	−0.17	0.07	−2.64 *
Gender (female vs. male)	−0.23	0.72	−0.32	−0.32	0.73	−0.43	−2.35	0.84	−2.81 **	−2.36	0.83	−2.84 **
Race/ethnicity (Black/Af. Am. vs. White)	3.67	0.95	3.87 ***	3.66	0.95	3.83 ***	1.22	1.14	1.07	1.18	1.15	1.03
Race/ethnicity- (other vs. White)	1.24	1.00	1.25	1.24	1.01	1.24	−1.75	1.04	−1.68	−1.75	1.05	−1.67
Education attainment	−0.27	0.47	−0.59	−0.31	0.48	−0.63	−0.20	0.55	−0.36	−0.15	0.55	−0.27
Personal annual income- ≥USD 10,009 v. ≤USD 10,008	−2.33	0.82	−2.84 **	−2.24	0.82	−2.72 **	−2.32	1.09	−2.12 *	−2.25	1.08	−2.08 *
Living arrangement (live w/others vs. live alone)	−0.60	0.73	−0.83	−0.59	0.72	−0.82	−0.12	0.82	−0.14	−0.10	0.82	−0.13
Years with disability	0.003	0.03	0.13	0.003	0.03	0.10	0.04	0.03	1.09	0.04	0.03	1.08
Self-rated physical health	−0.90	0.46	−1.97 *	−0.94	0.46	−2.03 *	−0.43	0.58	−0.73	−0.45	0.60	−0.76
Self-rated mental health	0.08	0.46	0.17	0.21	0.50	0.42	0.44	0.62	0.70	0.50	0.70	0.71
Physical function	0.15	0.05	3.15 **	0.15	0.05	3.15 **	0.25	0.05	5.39 ***	0.25	0.05	5.38 ***
AwD symptoms composite score	−4.61	0.41	−11.22 ***	*NA*			−5.08	0.60	−8.50 ***	*NA*		
Pain	*NA*			−0.18	0.05	−3.43 ***	*NA*			−0.16	0.06	−2.77 **
Fatigue	*NA*			−0.18	0.05	−3.47 ***	*NA*			−0.22	0.06	−3.67 **
Depression	*NA*			−0.21	0.05	−4.37 ***	*NA*			−0.21	0.06	−3.60 ***

Note: * *p* < 0.05; ** *p* < 0.01; *** *p* < 0.001; df1 = degrees of freedom in the numerator, df2 = degrees of freedom in the denominator; SE b = standard error of the b coefficient; gender (male = 0, female = 1), race/ethnicity (White = 1, African American/Black = 2, other categories = 3), personal income (≤USD 10,008 = 1, ≥USD 10,009 = 2), living arrangement (live alone = 1, live w/others = 2).

**Table 4:** Multivariate regression for satisfaction with social roles and activities at T1 and T3: AwD symptoms model comparisons.

	T1						T3					
	Composite AwD Symptoms			Individual AwD Symptoms			Composite AwD Symptoms			Individual AwD Symptoms		
Model Fit	*R^2^* 0.411 ***	*F*^(df1, df2)^ 26.08 ***^(12, 449)^		*R^2^* 0.414 ***	*F*^(df1, df2)^ 22.56 ***^(14, 447)^		*R^2^* 0.420 ***	*F*^(df1, df2)^ 17.68 ***^(12, 293)^		*R^2^* 0.427 ***	*F*^(df1, df2)^ 15.47 ***^(14, 291)^	
	*b*	*SE b*	*t*	*b*	*SE b*	*t*	*B*	*SE b*	*t*	*b*	*SE b*	*t*
Y intercept	41.79	4.48	9.32 ***	75.49	5.72	13.19 ***	47.22	5.48	8.61 ***	73.34	6.30	11.63 ***
Age (in years)	0.09	0.06	1.49	0.09	0.06	1.45	−0.10	0.08	−1.28	−0.10	0.08	−1.28
Gender (female vs. male)	−0.69	0.76	−0.91	−0.83	0.76	−1.06	−1.54	0.94	−1.63	−1.51	0.94	−1.60
Race/ethnicity (Black/Af. Am. vs. White)	2.33	1.00	2.34 *	2.22	1.00	2.23 *	0.72	1.19	0.60	0.63	1.21	0.52
Race/ethnicity (other vs. White)	−0.23	1.07	−0.22	−0.33	1.06	−0.31	−0.30	1.28	−0.23	−0.35	1.29	−0.27
Education attainment	−0.19	0.50	−0.37	−0.18	0.50	−0.36	−1.11	0.64	−1.72	−0.98	0.65	−1.51
Personal annual income (≥USD 10,009 vs. ≤USD 10,008)	−2.25	0.89	−2.51 *	−2.06	0.88	−2.34 *	−2.96	1.19	−2.49*	−2.71	1.18	−2.29 *
Living arrangement (live w/others vs. live alone)	−1.73	0.76	−2.26 *	−1.72	0.76	−2.25 *	0.88	0.91	0.97	0.91	0.91	0.99
Years with disability	0.03	0.03	1.15	0.03	0.03	1.09	0.08	0.04	2.18 *	0.08	0.04	2.20 *
Self-rated physical health	−1.41	0.55	−2.57 **	−1.54	0.54	−2.85 **	−0.80	0.63	−1.26	−0.84	0.63	−1.33
Self-rated mental health	−0.29	0.53	−0.55	−0.02	0.54	−0.04	−0.27	0.64	−0.41	−0.26	0.71	−0.36
Physical function	0.09	0.05	1.75	0.09	0.05	1.75	0.24	0.05	4.57 ***	0.23	0.05	4.52 ***
AwD symptoms composite score	−4.74	0.52	−9.15 ***	*NA*			−4.20	0.61	−6.86 ***	*NA*		
Pain	*NA*			−0.13	0.05	−2.62 **	*NA*			−0.06	0.07	0.95
Fatigue	*NA*			−0.20	0.06	−3.46 ***	*NA*			−0.26	0.06	−4.54 ***
Depression	*NA*			−0.25	0.05	−4.60 ***	*NA*			−0.16	0.07	−2.28 *

Note: * *p* < 0.05; ** *p* < 0.01; *** *p* < 0.001; df1 = degrees of freedom in the numerator, df2 = degrees of freedom in the denominator; SE b = standard error of the b coefficient; gender (male = 0, female = 1), race/ethnicity (White = 1, African American/Black = 2, other categories = 3), Personal income (≤USD 10,008 = 1, ≥USD 10,009 = 2), living arrangement (live alone = 1, live w/others = 2.

## Data Availability

The underlying data generated and analyzed during the current study cannot be sufficiently deidentified and, therefore, cannot be made publicly available due to ethical considerations. Deidentified data could be made available upon reasonable request for the purpose of further research via the corresponding author.

## Supplementary Materials

The following are available online at https://www.mdpi.com/article/10.3390/healthcare10050903/s1, Table S1: PROMIS Item Bank v2.0—Satisfaction with Social Roles and Activities; Table S2: PROMIS Item Bank v2.0—Ability to Participate in Social Roles and Activities; Table S3: PROMIS Item Bank v. 1.0—Emotional Distress—Depression; Table S4: PROMIS Bank v1.1—Pain Interference; Table S5: PROMIS Item Bank v.1.0—Fatigue; Table S6: PROMIS^®^ Item Bank v1.0—Physical Function with Mobility.
