# Supplementary material for: Aging with Disability Symptoms and Ability to Participate in, and Satisfaction with, Social Participation among Persons Aged 45–64
Source: Healthcare (Basel). 2022 May 13;10(5):903. doi: 10.3390/healthcare10050903 (PMC9141701; doi:10.3390/healthcare10050903)
Supplement: Supplementary file 1 [file healthcare-10-00903-s001.zip › healthcare-1685498-supplementary.pdf]

Aging with Disability Symptoms and Ability to Participate in, and Satisfaction with, Social Participation Among Persons Aged 45-64

Michelle Putnam [1,*], Kerri Morgan[2], Rachel Heeb [2], Yan Yan [3], Szu-Wei Chen [2] and Susan L. Stark [2]

PROMIS Item Bank v2.0 - Satisfaction with Social Roles and Activities

Satisfaction with Social Roles and Activities – Calibrated Items

Please respond to each item by marking one box per row.

		Not at all	A little bit	Somewhat	Quite a bit	Very much
SRPSAT17_CaPS	I am satisfied with the extent of my social activities outside my home	☐ 1	☐ 2	☐ 3	☐ 4	☐ 5
RP14_CaPS	I am satisfied with my current level of family activities......................................	☐ 1	☐ 2	☐ 3	☐ 4	☐ 5
RP7_CaPS	I am satisfied with my ability to maintain friendships...	☐ 1	☐ 2	☐ 3	☐ 4	☐ 5
SRPSAT03_CaPS	I am satisfied with how often I go out for entertainment	☐ 1	☐ 2	☐ 3	☐ 4	☐ 5
SRPSAT04_CaPS	I am satisfied with my ability to socialize with friends	☐ 1	☐ 2	☐ 3	☐ 4	☐ 5
SRPSAT05r1	I am satisfied with the amount of time I spend doing leisure activities.................	☐ 1	☐ 2	☐ 3	☐ 4	☐ 5
SRPSAT05_CaPS	I am satisfied with the amount of time I spend doing leisure activities with others	☐ 1	☐ 2	☐ 3	☐ 4	☐ 5
SRPSAT06r1	I am satisfied with my ability to do things for my family	☐ 1	☐ 2	☐ 3	☐ 4	☐ 5
SRPSAT07r1	I am satisfied with how much work I can do (include work at home).....................	☐ 1	☐ 2	☐ 3	☐ 4	☐ 5
SRPSAT08r1	I feel good about my ability to do things for my family	☐ 1	☐ 2	☐ 3	☐ 4	☐ 5
SRPSAT09r1	I am satisfied with my ability to do the work that is really important to me (include work at home)..........................	☐ 1	☐ 2	☐ 3	☐ 4	☐ 5
SRPSAT10r1	I am satisfied with my current level of social activity	☐ 1	☐ 2	☐ 3	☐ 4	☐ 5

© 2008-2012 PROMIS Health Organization and PROMIS Cooperative Group

		Not at all	A little bit	Somewhat	Quite a bit	Very much
SRPSAT15_ CaPS	I am satisfied with my ability to participate in family activities................	☐ 1	☐ 2	☐ 3	☐ 4	☐ 5
SRPSAT19r1	I am satisfied with my ability to do all of the community activities that are really important to me.....................................	☐ 1	☐ 2	☐ 3	☐ 4	☐ 5
SRPSAT20r1	I am satisfied with my ability to do things for my friends..............................	☐ 1	☐ 2	☐ 3	☐ 4	☐ 5
SRPSAT21r1	I am satisfied with the amount of time I spend doing work (include work at home)	☐ 1	☐ 2	☐ 3	☐ 4	☐ 5
SRPSAT22r1	I am happy with how much I do for my family..	☐ 1	☐ 2	☐ 3	☐ 4	☐ 5
SRPSAT23r1	I am satisfied with my ability to do leisure activities	☐ 1	☐ 2	☐ 3	☐ 4	☐ 5
SRPSAT23_ CaPS	I am satisfied with my ability to do leisure activities with others	☐ 1	☐ 2	☐ 3	☐ 4	☐ 5
SRPSAT24r1	I am satisfied with my ability to work (include work at home)..........................	☐ 1	☐ 2	☐ 3	☐ 4	☐ 5
SRPSAT25r1	I am satisfied with my current level of activities with my friends.......................	☐ 1	☐ 2	☐ 3	☐ 4	☐ 5
SRPSAT29_ CaPS	I am satisfied with my ability to engage in activities with friends.........................	☐ 1	☐ 2	☐ 3	☐ 4	☐ 5
SRPSAT33r1	I am satisfied with my ability to do things for fun outside my home	☐ 1	☐ 2	☐ 3	☐ 4	☐ 5
SRPSAT33_ CaPS	I am satisfied with my ability to do things for fun with others.......................	☐ 1	☐ 2	☐ 3	☐ 4	☐ 5
SRPSAT34r1	I feel good about my ability to do things for my friends...	☐ 1	☐ 2	☐ 3	☐ 4	☐ 5

© 2008-2012 PROMIS Health Organization and PROMIS Cooperative Group

		Not at all	A little bit	Somewhat	Quite a bit	Very much
SRPSAT35r1	The quality of my work is as good as I want it to be (include work at home)	☐ 1	☐ 2	☐ 3	☐ 4	☐ 5
SRPSAT36r1	I am happy with how much I do for my friends	☐ 1	☐ 2	☐ 3	☐ 4	☐ 5
SRPSAT37r1	I am satisfied with the amount of time I spend visiting friends	☐ 1	☐ 2	☐ 3	☐ 4	☐ 5
SRPSAT38r1	I am satisfied with the amount of time I spend performing my daily routines	☐ 1	☐ 2	☐ 3	☐ 4	☐ 5
SRPSAT38_CaPS	I am satisfied with the amount of time I spend doing things for my family...........	☐ 1	☐ 2	☐ 3	☐ 4	☐ 5
SRPSAT39r1	I am satisfied with my ability to do household chores/tasks	☐ 1	☐ 2	☐ 3	☐ 4	☐ 5
SRPSAT41_CaPS	I am satisfied with the extent of my social activities with groups of people....	☐ 1	☐ 2	☐ 3	☐ 4	☐ 5
SRPSAT43_CaPS	I am satisfied with my ability to keep in touch with others....................................	☐ 1	☐ 2	☐ 3	☐ 4	☐ 5
SRPSAT45_CaPS	I am satisfied with my ability to meet the needs of my family	☐ 1	☐ 2	☐ 3	☐ 4	☐ 5
SRPSAT46_CaPS	I am satisfied with my ability to meet the needs of my friends................................	☐ 1	☐ 2	☐ 3	☐ 4	☐ 5
SRPSAT47r1	I am satisfied with my ability to do regular personal and household responsibilities	☐ 1	☐ 2	☐ 3	☐ 4	☐ 5
SRPSAT47_CaPS	I am satisfied with my ability to do regular personal responsibilities	☐ 1	☐ 2	☐ 3	☐ 4	☐ 5
SRPSAT48r1	I am satisfied with my ability to do things for fun at home (like reading, listening to music, etc.)...........................	☐ 1	☐ 2	☐ 3	☐ 4	☐ 5

© 2008-2012 PROMIS Health Organization and PROMIS Cooperative Group

		Not at all	A little bit	Somewhat	Quite a bit	Very much
SRPSAT49r1	I am satisfied with my ability to perform my daily routines	☐ 1	☐ 2	☐ 3	☐ 4	☐ 5
SRPSAT50r1	I am satisfied with my ability to meet the needs of those who depend on me	☐ 1	☐ 2	☐ 3	☐ 4	☐ 5
SRPSAT51r1	I am satisfied with my ability to run errands	☐ 1	☐ 2	☐ 3	☐ 4	☐ 5
SRPSAT51_CaPS	I am satisfied with my ability to run errands for others	☐ 1	☐ 2	☐ 3	☐ 4	☐ 5
SRPSAT52r1	I am satisfied with my ability to do all of the leisure activities that are really important to me......................................	☐ 1	☐ 2	☐ 3	☐ 4	☐ 5
SRPSAT52_CaPS	I am satisfied with my ability to do all of the group activities that are really important to me......................................	☐ 1	☐ 2	☐ 3	☐ 4	☐ 5

© 2008-2012 PROMIS Health Organization and PROMIS Cooperative Group

PROMIS Item Bank v2.0 - Ability to Participate in Social Roles and Activities

Ability to Participate in Social Roles and Activities – Calibrated Items

Please respond to each item by marking one box per row.

		Never	Rarely	Sometimes	Usually	Always
RP1	I have trouble doing my regular daily work around the house	☐ 5	☐ 4	☐ 3	☐ 2	☐ 1
RP6	I have trouble meeting the needs of my friends	☐ 5	☐ 4	☐ 3	☐ 2	☐ 1
SRPPER_CaPS1	I have to limit social activities at home	☐ 5	☐ 4	☐ 3	☐ 2	☐ 1
SRPPER01r1	I have trouble meeting the needs of my family	☐ 5	☐ 4	☐ 3	☐ 2	☐ 1
SRPPER02r1	I am limited in doing my work (include work at home)	☐ 5	☐ 4	☐ 3	☐ 2	☐ 1
SRPPER03r1	I have to limit social activities outside my home	☐ 5	☐ 4	☐ 3	☐ 2	☐ 1
SRPPER04_CaPS	I have trouble participating in recreational activities with others	☐ 5	☐ 4	☐ 3	☐ 2	☐ 1
SRPPER05_CaPS	I have trouble doing everything for my family that I feel I should do	☐ 5	☐ 4	☐ 3	☐ 2	☐ 1
SRPPER06_CaPS	I have trouble accomplishing my usual work (include work at home)	☐ 5	☐ 4	☐ 3	☐ 2	☐ 1
SRPPER07_CaPS	I have trouble doing all of the family activities that I feel I should do	☐ 5	☐ 4	☐ 3	☐ 2	☐ 1
SRPPER08_CaPS	I have trouble doing all of the family activities that are really important to me.	☐ 5	☐ 4	☐ 3	☐ 2	☐ 1
SRPPER09_CaPS	I have trouble doing everything for work that I want to do (include work at home).	☐ 5	☐ 4	☐ 3	☐ 2	☐ 1

© 2008-2012 PROMIS Health Organization and PROMIS Cooperative Group

		Never	Rarely	Sometimes	Usually	Always
SRPPER11_CaPS	I have trouble doing all of my regular leisure activities with others	☐ 5	☐ 4	☐ 3	☐ 2	☐ 1
SRPPER13_CaPS	I have to limit social activities with groups of people......................................	☐ 5	☐ 4	☐ 3	☐ 2	☐ 1
SRPPER14r1	I have to limit my regular family activities	☐ 5	☐ 4	☐ 3	☐ 2	☐ 1
SRPPER15_CaPS	I have to limit the things I do for fun with others..	☐ 5	☐ 4	☐ 3	☐ 2	☐ 1
SRPPER16r1	I have to do my work for shorter periods of time than usual (include work at home)	☐ 5	☐ 4	☐ 3	☐ 2	☐ 1
SRPPER17r1	I feel limited in the amount of time I have for my family...................................	☐ 5	☐ 4	☐ 3	☐ 2	☐ 1
SRPPER18_CaPS	I have trouble doing all of the family activities that I want to do.........................	☐ 5	☐ 4	☐ 3	☐ 2	☐ 1
SRPPER20_CaPS	I have trouble doing all of the activities with friends that are really important to me..	☐ 5	☐ 4	☐ 3	☐ 2	☐ 1
SRPPER21_CaPS	I have trouble doing all the leisure activities with others that I want to do	☐ 5	☐ 4	☐ 3	☐ 2	☐ 1
SRPPER22_CaPS	I have trouble keeping up with my family responsibilities..............................	☐ 5	☐ 4	☐ 3	☐ 2	☐ 1
SRPPER23_CaPS	I have trouble doing all of my usual work (include work at home)	☐ 5	☐ 4	☐ 3	☐ 2	☐ 1
SRPPER26_CaPS	I have trouble doing all of the work that is really important to me (include work at home)	☐ 5	☐ 4	☐ 3	☐ 2	☐ 1
SRPPER28r1	I have to limit my regular activities with friends	☐ 5	☐ 4	☐ 3	☐ 2	☐ 1

© 2008-2012 PROMIS Health Organization and PROMIS Cooperative Group

		Never	Rarely	Sometimes	Usually	Always
SRPPER31_CaPS	I have trouble taking care of my regular personal responsibilities	☐ 5	☐ 4	☐ 3	☐ 2	☐ 1
SRPPER35_CaPS	I have trouble doing everything for my friends that I want to do	☐ 5	☐ 4	☐ 3	☐ 2	☐ 1
SRPPER36_CaPS	I have trouble doing all of the activities with friends that I feel I should do	☐ 5	☐ 4	☐ 3	☐ 2	☐ 1
SRPPER37_CaPS	I have trouble doing all of the work that I feel I should do (include work at home)	☐ 5	☐ 4	☐ 3	☐ 2	☐ 1
SRPPER42r1	I feel limited in my ability to visit friends	☐ 5	☐ 4	☐ 3	☐ 2	☐ 1
SRPPER43r1	I have trouble keeping in touch with others	☐ 5	☐ 4	☐ 3	☐ 2	☐ 1
SRPPER46_CaPS	I have trouble doing all of the activities with friends that I want to do	☐ 5	☐ 4	☐ 3	☐ 2	☐ 1
SRPPER47_CaPS	I have trouble keeping up with my work responsibilities (include work at home)	☐ 5	☐ 4	☐ 3	☐ 2	☐ 1
SRPPER54_CaPS	I have trouble doing everything for my friends that I feel I should do	☐ 5	☐ 4	☐ 3	☐ 2	☐ 1
SRPPER55r1	I feel limited in the amount of time I have to visit friends	☐ 5	☐ 4	☐ 3	☐ 2	☐ 1

© 2008-2012 PROMIS Health Organization and PROMIS Cooperative Group

Table S3: PROMIS Item Bank v. 1.0 – Emotional Distress – Depression

Emotional Distress - Depression – Calibrated Items

Please respond to each item by marking one box per row.

In the past 7 days...

		Never	Rarely	Sometimes	Often	Always
EDDEP04	I felt worthless………………………..	☐ 1	☐ 2	☐ 3	☐ 4	☐ 5
EDDEP05	I felt that I had nothing to look forward to	☐ 1	☐ 2	☐ 3	☐ 4	☐ 5
EDDEP06	I felt helpless……………………………	☐ 1	☐ 2	☐ 3	☐ 4	☐ 5
EDDEP07	I withdrew from other people…………..	☐ 1	☐ 2	☐ 3	☐ 4	☐ 5
EDDEP09	I felt that nothing could cheer me up…	☐ 1	☐ 2	☐ 3	☐ 4	☐ 5
EDDEP14	I felt that I was not as good as other people………………………………..	☐ 1	☐ 2	☐ 3	☐ 4	☐ 5
EDDEP17	I felt sad………………………………	☐ 1	☐ 2	☐ 3	☐ 4	☐ 5
EDDEP19	I felt that I wanted to give up on everything…………………………….	☐ 1	☐ 2	☐ 3	☐ 4	☐ 5
EDDEP21	I felt that I was to blame for things……..	☐ 1	☐ 2	☐ 3	☐ 4	☐ 5

© 2008-2016 PROMIS Health Organization and PROMIS Cooperative Group

In the past 7 days…

		Never	Rarely	Sometimes	Often	Always
EDDEP22	I felt like a failure……………………	☐ 1	☐ 2	☐ 3	☐ 4	☐ 5
EDDEP23	I had trouble feeling close to people……	☐ 1	☐ 2	☐ 3	☐ 4	☐ 5
EDDEP26	I felt disappointed in myself………….	☐ 1	☐ 2	☐ 3	☐ 4	☐ 5
EDDEP27	I felt that I was not needed…………...	☐ 1	☐ 2	☐ 3	☐ 4	☐ 5
EDDEP28	I felt lonely…………………………...	☐ 1	☐ 2	☐ 3	☐ 4	☐ 5
EDDEP29	I felt depressed………………………….	☐ 1	☐ 2	☐ 3	☐ 4	☐ 5
EDDEP30	I had trouble making decisions……….	☐ 1	☐ 2	☐ 3	☐ 4	☐ 5
EDDEP31	I felt discouraged about the future……	☐ 1	☐ 2	☐ 3	☐ 4	☐ 5
EDDEP35	I found that things in my life were overwhelming………………………...	☐ 1	☐ 2	☐ 3	☐ 4	☐ 5
EDDEP36	I felt unhappy…………………………...	☐ 1	☐ 2	☐ 3	☐ 4	☐ 5
EDDEP39	I felt I had no reason for living……….	☐ 1	☐ 2	☐ 3	☐ 4	☐ 5

© 2008-2016 PROMIS Health Organization and PROMIS Cooperative Group

In the past 7 days…

		Never	Rarely	Sometimes	Often	Always
EDDEP41	I felt hopeless…………………………..	☐ 1	☐ 2	☐ 3	☐ 4	☐ 5
EDDEP42	I felt ignored by people………………..	☐ 1	☐ 2	☐ 3	☐ 4	☐ 5
EDDEP44	I felt upset for no reason………………	☐ 1	☐ 2	☐ 3	☐ 4	☐ 5
EDDEP45	I felt that nothing was interesting……..	☐ 1	☐ 2	☐ 3	☐ 4	☐ 5
EDDEP46	I felt pessimistic……………………….	☐ 1	☐ 2	☐ 3	☐ 4	☐ 5
EDDEP48	I felt that my life was empty…………..	☐ 1	☐ 2	☐ 3	☐ 4	☐ 5
EDDEP50	I felt guilty…………………………….	☐ 1	☐ 2	☐ 3	☐ 4	☐ 5
EDDEP54	I felt emotionally exhausted…………..	☐ 1	☐ 2	☐ 3	☐ 4	☐ 5

© 2008-2016 PROMIS Health Organization and PROMIS Cooperative Group

Table S4: PROMIS Bank v1.1 – Pain Interference

Pain Interference

Please respond to each item by marking one box per row.

In the past 7 days…

		Not at all	A little bit	Somewhat	Quite a bit	Very much
PAININ1	How difficult was it for you to take in new information because of pain?	☐ 1	☐ 2	☐ 3	☐ 4	☐ 5
PAININ3	How much did pain interfere with your enjoyment of life?	☐ 1	☐ 2	☐ 3	☐ 4	☐ 5
PAININ5	How much did pain interfere with your ability to participate in leisure activities?...................	☐ 1	☐ 2	☐ 3	☐ 4	☐ 5
PAININ6	How much did pain interfere with your close personal relationships?....................................	☐ 1	☐ 2	☐ 3	☐ 4	☐ 5
PAININ8	How much did pain interfere with your ability to concentrate?..	☐ 1	☐ 2	☐ 3	☐ 4	☐ 5
PAININ9	How much did pain interfere with your day to day activities?..	☐ 1	☐ 2	☐ 3	☐ 4	☐ 5
PAININ10	How much did pain interfere with your enjoyment of recreational activities?...............	☐ 1	☐ 2	☐ 3	☐ 4	☐ 5

©2008-2013 PROMIS Health Organization and PROMIS Cooperative Group

In the past 7 days…

		Not at all	A little bit	Somewhat	Quite a bit	Very much
PAININ11	How often did you feel emotionally tense because of your pain?..	☐ 1	☐ 2	☐ 3	☐ 4	☐ 5
PAININ12	How much did pain interfere with the things you usually do for fun?	☐ 1	☐ 2	☐ 3	☐ 4	☐ 5
PAININ13	How much did pain interfere with your family life?..	☐ 1	☐ 2	☐ 3	☐ 4	☐ 5
PAININ17	How much did pain interfere with your relationships with other people?	☐ 1	☐ 2	☐ 3	☐ 4	☐ 5
PAININ18	How much did pain interfere with your ability to work (include work at home)?	☐ 1	☐ 2	☐ 3	☐ 4	☐ 5
PAININ19	How much did pain make it difficult to fall asleep?...	☐ 1	☐ 2	☐ 3	☐ 4	☐ 5
PAININ20	How much did pain feel like a burden to you?..	☐ 1	☐ 2	☐ 3	☐ 4	☐ 5
PAININ22	How much did pain interfere with work around the home?...	☐ 1	☐ 2	☐ 3	☐ 4	☐ 5

©2008-2013 PROMIS Health Organization and PROMIS Cooperative Group

In the past 7 days…

		Not at all	A little bit	Somewhat	Quite a bit	Very much
PAININ31	How much did pain interfere with your ability to participate in social activities?	☐ 1	☐ 2	☐ 3	☐ 4	☐ 5
PAININ34	How much did pain interfere with your household chores?..	☐ 1	☐ 2	☐ 3	☐ 4	☐ 5
PAININ35	How much did pain interfere with your ability to make trips from home that kept you gone for more than 2 hours?...	☐ 1	☐ 2	☐ 3	☐ 4	☐ 5
PAININ36	How much did pain interfere with your enjoyment of social activities?..........................	☐ 1	☐ 2	☐ 3	☐ 4	☐ 5
PAININ48	How much did pain interfere with your ability to do household chores?...................................	☐ 1	☐ 2	☐ 3	☐ 4	☐ 5
PAININ49	How much did pain interfere with your ability to remember things?...	☐ 1	☐ 2	☐ 3	☐ 4	☐ 5
PAININ56	How irritable did you feel because of pain?..	☐ 1	☐ 2	☐ 3	☐ 4	☐ 5
PAININ14	How much did pain interfere with doing your tasks away from home (e.g., getting groceries, running errands)?..	☐ 1	☐ 2	☐ 3	☐ 4	☐ 5

©2008-2013 PROMIS Health Organization and PROMIS Cooperative Group

In the past 7 days…

		Never	Rarely	Sometimes	Often	Always
PAININ16	How often did pain make you feel depressed?..	☐ 1	☐ 2	☐ 3	☐ 4	☐ 5
PAININ24	How often was pain distressing to you?	☐ 1	☐ 2	☐ 3	☐ 4	☐ 5
PAININ26	How often did pain keep you from socializing with others?...	☐ 1	☐ 2	☐ 3	☐ 4	☐ 5
PAININ29	How often was your pain so severe you could think of nothing else?...	☐ 1	☐ 2	☐ 3	☐ 4	☐ 5
PAININ32	How often did pain make you feel discouraged?	☐ 1	☐ 2	☐ 3	☐ 4	☐ 5
PAININ37	How often did pain make you feel anxious?	☐ 1	☐ 2	☐ 3	☐ 4	☐ 5
PAININ38	How often did you avoid social activities because it might make you hurt more?	☐ 1	☐ 2	☐ 3	☐ 4	☐ 5
PAININ40	How often did pain prevent you from walking more than 1 mile?...	☐ 1	☐ 2	☐ 3	☐ 4	☐ 5
PAININ42	How often did pain prevent you from standing for more than one hour?.....................................	☐ 1	☐ 2	☐ 3	☐ 4	☐ 5

©2008-2013 PROMIS Health Organization and PROMIS Cooperative Group

In the past 7 days…

		Never	Rarely	Sometimes	Often	Always
PAININ46	How often did pain make it difficult for you to plan social activities?	☐ 1	☐ 2	☐ 3	☐ 4	☐ 5
PAININ47	How often did pain prevent you from standing for more than 30 minutes?	☐ 1	☐ 2	☐ 3	☐ 4	☐ 5
PAININ50	How often did pain prevent you from sitting for more than 30 minutes?	☐ 1	☐ 2	☐ 3	☐ 4	☐ 5
PAININ51	How often did pain prevent you from sitting for more than 10 minutes?	☐ 1	☐ 2	☐ 3	☐ 4	☐ 5
PAININ52	How often was it hard to plan social activities because you didn't know if you would be in pain?	☐ 1	☐ 2	☐ 3	☐ 4	☐ 5
PAININ53	How often did pain restrict your social life to your home?	☐ 1	☐ 2	☐ 3	☐ 4	☐ 5
PAININ55	How often did pain prevent you from sitting for more than one hour?	☐ 1	☐ 2	☐ 3	☐ 4	☐ 5

In the past 7 days…

		Never	Once a week or less	Once every few days	Once a day	Every few hours
PAININ54	How often did pain keep you from getting into a standing position?	☐ 1	☐ 2	☐ 3	☐ 4	☐ 5

©2008-2013 PROMIS Health Organization and PROMIS Cooperative Group

Fatigue – Calibrated Items

Please respond to each item by marking one box per row.

In the past 7 days...

		Never	Rarely	Sometimes	Often	Always
FATEXP2	How often did you feel run-down?...........	☐ 1	☐ 2	☐ 3	☐ 4	☐ 5
FATEXP5	How often did you experience extreme exhaustion?..	☐ 1	☐ 2	☐ 3	☐ 4	☐ 5
FATEXP6	How often did you feel tired even when you hadn't done anything?........................	☐ 1	☐ 2	☐ 3	☐ 4	☐ 5
FATEXP7	How often did you feel your fatigue was beyond your control?...............................	☐ 1	☐ 2	☐ 3	☐ 4	☐ 5
FATEXP16	How often were you sluggish?.................	☐ 1	☐ 2	☐ 3	☐ 4	☐ 5
FATEXP18	How often did you run out of energy?..	☐ 1	☐ 2	☐ 3	☐ 4	☐ 5
FATEXP19	How often were you physically drained?...	☐ 1	☐ 2	☐ 3	☐ 4	☐ 5
FATEXP20	How often did you feel tired?..................	☐ 1	☐ 2	☐ 3	☐ 4	☐ 5

© 2008-2017 PROMIS Health Organization and PROMIS Cooperative Group

In the past 7 days...

		Never	Rarely	Sometimes	Often	Always
FATEXP22	How often were you bothered by your fatigue?..	☐ 1	☐ 2	☐ 3	☐ 4	☐ 5
FATEXP24	How often did you have enough energy to enjoy the things you do for fun?...........	☐ 5	☐ 4	☐ 3	☐ 2	☐ 1
FATEXP26	How often were you too tired to enjoy life?..	☐ 1	☐ 2	☐ 3	☐ 4	☐ 5
FATEXP28	How often were you too tired to feel happy?...	☐ 1	☐ 2	☐ 3	☐ 4	☐ 5
FATEXP29	How often did you feel totally drained?....	☐ 1	☐ 2	☐ 3	☐ 4	☐ 5
FATEXP31	How often were you energetic?.................	☐ 5	☐ 4	☐ 3	☐ 2	☐ 1
FATEXP48	How often did you find yourself getting tired easily?..	☐ 1	☐ 2	☐ 3	☐ 4	☐ 5
FATEXP49	How often did you think about your fatigue?..	☐ 1	☐ 2	☐ 3	☐ 4	☐ 5
FATEXP54	How often did you have physical energy?..	☐ 5	☐ 4	☐ 3	☐ 2	☐ 1

© 2008-2017 PROMIS Health Organization and PROMIS Cooperative Group

In the past 7 days....

		Never	Rarely	Sometimes	Often	Always
FATIMP3	How often did you have to push yourself to get things done because of your fatigue?..	☐ 1	☐ 2	☐ 3	☐ 4	☐ 5
FATIMP4	How often did your fatigue interfere with your social activities?................................	☐ 1	☐ 2	☐ 3	☐ 4	☐ 5
FATIMP5	How often were you less effective at work due to your fatigue (include work at home)?..	☐ 1	☐ 2	☐ 3	☐ 4	☐ 5
FATIMP6	How often did your fatigue make you feel slowed down in your thinking?..........	☐ 1	☐ 2	☐ 3	☐ 4	☐ 5
FATIMP8	How often were you too tired to watch television? …………………………….	☐ 1	☐ 2	☐ 3	☐ 4	☐ 5
FATIMP9	How often did your fatigue make it difficult to plan activities ahead of time?...	☐ 1	☐ 2	☐ 3	☐ 4	☐ 5
FATIMP10	How often did your fatigue make it difficult to start anything new?................	☐ 1	☐ 2	☐ 3	☐ 4	☐ 5
FATIMP11	How often did your fatigue make you more forgetful?..	☐ 1	☐ 2	☐ 3	☐ 4	☐ 5

© 2008-2017 PROMIS Health Organization and PROMIS Cooperative Group

In the past 7 days...

		Never	Rarely	Sometimes	Often	Always
FATIMP13	How often were you too tired to do errands?..	☐ 1	☐ 2	☐ 3	☐ 4	☐ 5
FATIMP14	How often did your fatigue make it difficult to organize your thoughts when doing things at work (include work at home)?...	☐ 1	☐ 2	☐ 3	☐ 4	☐ 5
FATIMP15	How often did your fatigue interfere with your ability to engage in recreational activities?..	☐ 1	☐ 2	☐ 3	☐ 4	☐ 5
FATIMP16	How often did you have trouble finishing things because of your fatigue?…………	☐ 1	☐ 2	☐ 3	☐ 4	☐ 5
FATIMP17	How often did your fatigue make it difficult to make decisions?.....................	☐ 1	☐ 2	☐ 3	☐ 4	☐ 5
FATIMP18	How often did you have to limit your social activities because of your fatigue?...	☐ 1	☐ 2	☐ 3	☐ 4	☐ 5

© 2008-2017 PROMIS Health Organization and PROMIS Cooperative Group

In the past 7 days...

		Never	Rarely	Sometimes	Often	Always
FATIMP19	How often were you too tired to do your household chores?....................................	☐ 1	☐ 2	☐ 3	☐ 4	☐ 5
FATIMP20	How often did your fatigue make you feel less alert?...	☐ 1	☐ 2	☐ 3	☐ 4	☐ 5
FATIMP21	How often were you too tired to take a bath or shower?..	☐ 1	☐ 2	☐ 3	☐ 4	☐ 5
FATIMP22	How often did your fatigue make it difficult to organize your thoughts when doing things at home?..............................	☐ 1	☐ 2	☐ 3	☐ 4	☐ 5
FATIMP24	How often did you have trouble starting things because of your fatigue?................	☐ 1	☐ 2	☐ 3	☐ 4	☐ 5
FATIMP25	How often was it an effort to carry on a conversation because of your fatigue?...	☐ 1	☐ 2	☐ 3	☐ 4	☐ 5
FATIMP26	How often were you too tired to socialize with your family?.....................................	☐ 1	☐ 2	☐ 3	☐ 4	☐ 5
FATIMP29	How often were you too tired to leave the house?...	☐ 1	☐ 2	☐ 3	☐ 4	☐ 5

© 2008-2017 PROMIS Health Organization and PROMIS Cooperative Group

In the past 7 days...

		Never	Rarely	Sometimes	Often	Always
FATIMP30	How often were you too tired to think clearly?..	☐ 1	☐ 2	☐ 3	☐ 4	☐ 5
FATIMP33	How often did your fatigue limit you at work (include work at home)?.................	☐ 1	☐ 2	☐ 3	☐ 4	☐ 5
FATIMP40	How often did you have enough energy to exercise strenuously? ………………	☐ 5	☐ 4	☐ 3	☐ 2	☐ 1
FATIMP42	How often were you less effective at home due to your fatigue?........................	☐ 1	☐ 2	☐ 3	☐ 4	☐ 5
FATIMP53	How often were you too tired to take a short walk?...	☐ 1	☐ 2	☐ 3	☐ 4	☐ 5
FATIMP55	How often did you have to force yourself to get up and do things because of your fatigue?..	☐ 1	☐ 2	☐ 3	☐ 4	☐ 5
FATIMP56	How often were you too tired to socialize with your friends?...................................	☐ 1	☐ 2	☐ 3	☐ 4	☐ 5

© 2008-2017 PROMIS Health Organization and PROMIS Cooperative Group

During the past 7 days...

		Not at all	A little bit	Somewhat	Quite a bit	Very much
AN1	I feel listless ("washed out")…………...	☐ 1	☐ 2	☐ 3	☐ 4	☐ 5
AN2	I feel tired…………………………...	☐ 1	☐ 2	☐ 3	☐ 4	☐ 5
AN3	I have trouble starting things because I am tired…………………………………	☐ 1	☐ 2	☐ 3	☐ 4	☐ 5
AN4	I have trouble finishing things because I am tired……………………………...	☐ 1	☐ 2	☐ 3	☐ 4	☐ 5
AN5	I have energy…………………………..	☐ 5	☐ 4	☐ 3	☐ 2	☐ 1
AN7	I am able to do my usual activities…...….	☐ 5	☐ 4	☐ 3	☐ 2	☐ 1
AN8	I need to sleep during the day……...……	☐ 1	☐ 2	☐ 3	☐ 4	☐ 5
AN12	I am too tired to eat……………………	☐ 1	☐ 2	☐ 3	☐ 4	☐ 5
AN14	I need help doing my usual activities……	☐ 1	☐ 2	☐ 3	☐ 4	☐ 5
AN15	I am frustrated by being too tired to do the things I want to do………………..…	☐ 1	☐ 2	☐ 3	☐ 4	☐ 5

© 2008-2017 PROMIS Health Organization and PROMIS Cooperative Group

During the past 7 days...

		Not at all	A little bit	Somewhat	Quite a bit	Very much
AN16	I have to limit my social activity because I am tired………………………………..	☐ 1	☐ 2	☐ 3	☐ 4	☐ 5
	**In the past 7 days…**					
FATEXP12	To what degree did you feel tired even when you hadn't done anything?...............	☐ 1	☐ 2	☐ 3	☐ 4	☐ 5
FATEXP13	How bushed were you on average?...........	☐ 1	☐ 2	☐ 3	☐ 4	☐ 5
FATEXP21	How fatigued were you when your fatigue was at its worst?...........................	☐ 1	☐ 2	☐ 3	☐ 4	☐ 5
FATEXP34	How tired did you feel on average?..........	☐ 1	☐ 2	☐ 3	☐ 4	☐ 5
FATEXP35	How much were you bothered by your fatigue on average?..................................	☐ 1	☐ 2	☐ 3	☐ 4	☐ 5
FATEXP36	How exhausted were you on average?......	☐ 1	☐ 2	☐ 3	☐ 4	☐ 5
FATEXP38	How fatigued were you on the day you felt most fatigued?...................................	☐ 1	☐ 2	☐ 3	☐ 4	☐ 5

© 2008-2017 PROMIS Health Organization and PROMIS Cooperative Group

In the past 7 days...

		Not at all	A little bit	Somewhat	Quite a bit	Very much
FATEXP40	How fatigued were you on average?.........	☐ 1	☐ 2	☐ 3	☐ 4	☐ 5
FATEXP41	How run-down did you feel on average?...	☐ 1	☐ 2	☐ 3	☐ 4	☐ 5
FATEXP42	How much mental energy did you have on average?..	☐ 5	☐ 4	☐ 3	☐ 2	☐ 1
FATEXP43	How physically drained were you on average?...	☐ 1	☐ 2	☐ 3	☐ 4	☐ 5
FATEXP44	How energetic were you on average?......	☐ 5	☐ 4	☐ 3	☐ 2	☐ 1
FATEXP45	How sluggish were you on average?........	☐ 1	☐ 2	☐ 3	☐ 4	☐ 5
FATEXP50	How fatigued were you on the day you felt least fatigued?....................................	☐ 1	☐ 2	☐ 3	☐ 4	☐ 5
FATEXP51	How easily did you find yourself getting tired on average?......................................	☐ 1	☐ 2	☐ 3	☐ 4	☐ 5
FATEXP52	How wiped out were you on average?.....	☐ 1	☐ 2	☐ 3	☐ 4	☐ 5

© 2008-2017 PROMIS Health Organization and PROMIS Cooperative Group

In the past 7 days...

		Not at all	A little bit	Somewhat	Quite a bit	Very much
FATIMP1	To what degree did you have to push yourself to get things done because of your fatigue?...	☐ 1	☐ 2	☐ 3	☐ 4	☐ 5
FATIMP2	To what degree did your fatigue make you feel slowed down in your thinking?...	☐ 1	☐ 2	☐ 3	☐ 4	☐ 5
FATIMP27	To what degree did you have trouble starting things because of your fatigue?...	☐ 1	☐ 2	☐ 3	☐ 4	☐ 5
FATIMP28	How hard was it for you to carry on a conversation because of your fatigue?....	☐ 1	☐ 2	☐ 3	☐ 4	☐ 5
FATIMP34	To what degree did you have to limit your social activities because of your fatigue?...	☐ 1	☐ 2	☐ 3	☐ 4	☐ 5
FATIMP35	To what degree did your fatigue make it difficult to organize your thoughts when doing things at home?..............................	☐ 1	☐ 2	☐ 3	☐ 4	☐ 5
FATIMP36	To what degree did your fatigue make it difficult to start anything new?................	☐ 1	☐ 2	☐ 3	☐ 4	☐ 5

© 2008-2017 PROMIS Health Organization and PROMIS Cooperative Group

In the past 7 days...

		Not at all	A little bit	Somewhat	Quite a bit	Very much
FATIMP37	Due to your fatigue were you less effective at work (include work at home)?...	☐ 1	☐ 2	☐ 3	☐ 4	☐ 5
FATIMP38	To what degree did your fatigue make it difficult to make decisions?......................	☐ 1	☐ 2	☐ 3	☐ 4	☐ 5
FATIMP43	To what degree did your fatigue make it difficult to organize your thoughts when doing things at work (include work at home)?...	☐ 1	☐ 2	☐ 3	☐ 4	☐ 5
FATIMP44	To what degree did your fatigue make you more forgetful?.................................	☐ 1	☐ 2	☐ 3	☐ 4	☐ 5
FATIMP45	To what degree did your fatigue interfere with your ability to engage in recreational activities?..............................	☐ 1	☐ 2	☐ 3	☐ 4	☐ 5
FATIMP47	To what degree did you have to force yourself to get up and do things because of your fatigue?..	☐ 1	☐ 2	☐ 3	☐ 4	☐ 5
FATIMP48	To what degree did your fatigue interfere with your social activities?......................	☐ 1	☐ 2	☐ 3	☐ 4	☐ 5

© 2008-2017 PROMIS Health Organization and PROMIS Cooperative Group

In the past 7 days...

		Not at all	A little bit	Somewhat	Quite a bit	Very much
FATIMP49	To what degree did your fatigue interfere with your physical functioning?..............	☐ 1	☐ 2	☐ 3	☐ 4	☐ 5
FATIMP50	Did fatigue make you less effective at home?...	☐ 1	☐ 2	☐ 3	☐ 4	☐ 5
FATIMP51	To what degree did you have trouble finishing things because of your fatigue?...	☐ 1	☐ 2	☐ 3	☐ 4	☐ 5
FATIMP52	To what degree did your fatigue make you feel less alert?..................................	☐ 1	☐ 2	☐ 3	☐ 4	☐ 5

During the past 7 days...

		Not at all	A little bit	Somewhat	Quite a bit	Very much
HI7	I feel fatigued……………..…………….	☐ 1	☐ 2	☐ 3	☐ 4	☐ 5
HI12	I feel weak all over ………..……………	☐ 1	☐ 2	☐ 3	☐ 4	☐ 5

In the past 7 days…

		None	1 day	2-3 days	4-5 days	6-7 days
FATEXP46	On how many days was your fatigue worse in the morning?............................	☐ 1	☐ 2	☐ 3	☐ 4	☐ 5

		None	Mild	Moderate	Severe	Very severe
FATEXP56	What was the level of your fatigue on most days?...	☐ 1	☐ 2	☐ 3	☐ 4	☐ 5

© 2008-2017 PROMIS Health Organization and PROMIS Cooperative Group

PROMIS Physical Function with Mobility

Please respond to each item by marking one box per row.

The following questions ask about your ability to stand and move with and without support. "Support" means using items such as canes, walking sticks, walkers and leg braces, or other people.		**Yes**	**No**			
PF_Screener1	Can you stand (with or without support)?	☐	☐			
		Yes→ proceed to PF_Screener2<br>No→ proceed to CAT with items only from Group A				
The following questions ask about your ability to stand and move with and without support. "Support" means using items such as canes, walking sticks, walkers and leg braces, or other people.		**Yes**	**No**			
PF_Screener2	Can you walk 25 feet on a level surface (with or without support)?	☐	☐			
		Yes→ proceed to CAT with all items (Groups A, B, and C)<br>No→ proceed to CAT with items only from Groups A and C				
	**Group A Items (PF_1 thru PFC53)**	**Without any difficulty**	**With a little difficulty**	**With some difficulty**	**With much difficulty**	**Unable to do**
PF_1	Are you able to go OUTSIDE the home, for example to shop or visit a doctor's office?	☐ 5	☐ 4	☐ 3	☐ 2	☐ 1
PF_10	Are you able to dial a number on a phone with large buttons?....................................	☐ 5	☐ 4	☐ 3	☐ 2	☐ 1
PF_11	Are you able to clean up after a meal?.......	☐ 5	☐ 4	☐ 3	☐ 2	☐ 1
PF_12	How much difficulty do you currently have opening previously opened jars?	☐ 5	☐ 4	☐ 3	☐ 2	☐ 1
PF_13	Are you able to dial a number on the keypad of a cell phone?	☐ 5	☐ 4	☐ 3	☐ 2	☐ 1
PF_14	Are you able to roll onto your stomach while lying in bed?...................................	☐ 5	☐ 4	☐ 3	☐ 2	☐ 1

© 2008-2016 PROMIS Health Organization and PROMIS Cooperative Group

		Without any difficulty	With a little difficulty	With some difficulty	With much difficulty	Unable to do
PF_15	Are you able to pull up covers while lying in bed?	☐ 5	☐ 4	☐ 3	☐ 2	☐ 1
PF_18	Are you able to open and squeeze a new tube of toothpaste?	☐ 5	☐ 4	☐ 3	☐ 2	☐ 1
PF_19	How much difficulty do you currently have pulling up and fastening your pants after a bowel movement?	☐ 5	☐ 4	☐ 3	☐ 2	☐ 1
PF_2	Are you able to fasten and unfasten a seatbelt?	☐ 5	☐ 4	☐ 3	☐ 2	☐ 1
PF_20	Are you able to do chores such as vacuuming carpet or mopping floors?	☐ 5	☐ 4	☐ 3	☐ 2	☐ 1
PF_22	Are you able to lift small objects (such as a can of soup) above your shoulder?	☐ 5	☐ 4	☐ 3	☐ 2	☐ 1
PF_23	Are you able to reach and get down an object (such as a can of soup) from above your head?	☐ 5	☐ 4	☐ 3	☐ 2	☐ 1
PF_24	Are you able to carry a bag of groceries for a short distance?	☐ 5	☐ 4	☐ 3	☐ 2	☐ 1
PF_25	Are you able to put on socks?	☐ 5	☐ 4	☐ 3	☐ 2	☐ 1
PF_26	Are you able to pull a sweater or t-shirt over your head?	☐ 5	☐ 4	☐ 3	☐ 2	☐ 1
PF_27	How much difficulty do you currently have cleaning yourself after a bowel movement?	☐ 5	☐ 4	☐ 3	☐ 2	☐ 1
PF_28	Are you able to do an exercise of your choice for 20 minutes several times a week?	☐ 5	☐ 4	☐ 3	☐ 2	☐ 1

© 2008-2016 PROMIS Health Organization and PROMIS Cooperative Group

		Without any difficulty	With a little difficulty	With some difficulty	With much difficulty	Unable to do
PF_29	Are you able to do strenuous tasks around the house like cleaning the bathroom or scrubbing floors?	☐ 5	☐ 4	☐ 3	☐ 2	☐ 1
PF_30	Are you able to pour liquid from a container into a cup?	☐ 5	☐ 4	☐ 3	☐ 2	☐ 1
PF_31	Are you able to turn pages in a book?	☐ 5	☐ 4	☐ 3	☐ 2	☐ 1
PF_32	Are you able to prepare simple meals for yourself or for others?	☐ 5	☐ 4	☐ 3	☐ 2	☐ 1
PF_33	Are you able to reach above your head?	☐ 5	☐ 4	☐ 3	☐ 2	☐ 1
PF_34	Are you able to do housework like vacuuming or sweeping floors?	☐ 5	☐ 4	☐ 3	☐ 2	☐ 1
PF_35	Are you able to do yard work like raking leaves or sweeping the driveway?	☐ 5	☐ 4	☐ 3	☐ 2	☐ 1
PF_36	Are you able to take a letter out of an envelope?	☐ 5	☐ 4	☐ 3	☐ 2	☐ 1
PF_37	Are you able to push the buttons on a television remote control?	☐ 5	☐ 4	☐ 3	☐ 2	☐ 1
PF_38	Are you able to get items in and out of a wallet?	☐ 5	☐ 4	☐ 3	☐ 2	☐ 1
PF_39	Are you able to turn a key to unlock a door?	☐ 5	☐ 4	☐ 3	☐ 2	☐ 1
PF_4	Are you able to take a shower?	☐ 5	☐ 4	☐ 3	☐ 2	☐ 1
PF_40	Are you able to turn sink faucets on and off?	☐ 5	☐ 4	☐ 3	☐ 2	☐ 1

© 2008-2016 PROMIS Health Organization and PROMIS Cooperative Group

		Without any difficulty	With a little difficulty	With some difficulty	With much difficulty	Unable to do
PF_41	Are you able to type a few sentences on a computer keyboard?	☐ 5	☐ 4	☐ 3	☐ 2	☐ 1
PF_42	Are you able to move about the house?	☐ 5	☐ 4	☐ 3	☐ 2	☐ 1
PF_43	Are you able to use a regular computer mouse?	☐ 5	☐ 4	☐ 3	☐ 2	☐ 1
PF_44	Are you able to move wet clothes from the washer to the dryer?	☐ 5	☐ 4	☐ 3	☐ 2	☐ 1
PF_45	Are you able to move around on a slippery surface outdoors?	☐ 5	☐ 4	☐ 3	☐ 2	☐ 1
PF_46	Are you able to move about in a dark room or hallway without falling?	☐ 5	☐ 4	☐ 3	☐ 2	☐ 1
PF_47	Are you able to do vigorous activities, such as playing sports?	☐ 5	☐ 4	☐ 3	☐ 2	☐ 1
PF_48	Are you able to carry a shopping bag?	☐ 5	☐ 4	☐ 3	☐ 2	☐ 1
PF_49	Are you able to dress yourself?	☐ 5	☐ 4	☐ 3	☐ 2	☐ 1
PF_5	Are you able to wash and dry your feet?	☐ 5	☐ 4	☐ 3	☐ 2	☐ 1
PF_50	Are you able to wash dishes and utensils by hand?	☐ 5	☐ 4	☐ 3	☐ 2	☐ 1
PF_51	How much difficulty do you currently have sitting down on a low, soft couch?	☐ 5	☐ 4	☐ 3	☐ 2	☐ 1
PF_54	Are you able to transfer from a bed to a chair or a wheelchair?	☐ 5	☐ 4	☐ 3	☐ 2	☐ 1

© 2008-2016 PROMIS Health Organization and PROMIS Cooperative Group

		Without any difficulty	With a little difficulty	With some difficulty	With much difficulty	Unable to do
PF_55	If you need to, are you physically able to rush for a short distance, such as to cross a street?	☐ 5	☐ 4	☐ 3	☐ 2	☐ 1
PF_56	Are you able to move from the street to the sidewalk without a curb cut?	☐ 5	☐ 4	☐ 3	☐ 2	☐ 1
PF_57	Are you able to use a moving escalator safely?	☐ 5	☐ 4	☐ 3	☐ 2	☐ 1
PF_58	Are you able to fasten buttons on a shirt or blouse?	☐ 5	☐ 4	☐ 3	☐ 2	☐ 1
PF_59	Are you able to hold a card or letter in order to read it?	☐ 5	☐ 4	☐ 3	☐ 2	☐ 1
PF_6	Are you able to cut your toenails?	☐ 5	☐ 4	☐ 3	☐ 2	☐ 1
PF_60	Are you able to fold clean laundry?	☐ 5	☐ 4	☐ 3	☐ 2	☐ 1
PF_61	Are you able to get into a vehicle?	☐ 5	☐ 4	☐ 3	☐ 2	☐ 1
PF_62	How much difficulty do you currently have getting into and out of a truck, bus, shuttle van or sport utility vehicle?	☐ 5	☐ 4	☐ 3	☐ 2	☐ 1
PF_63	How much difficulty do you currently have applying spreads to breads using a knife?	☐ 5	☐ 4	☐ 3	☐ 2	☐ 1
PF_64	How much difficulty do you currently have playing cards or Bingo or other light recreational activities?	☐ 5	☐ 4	☐ 3	☐ 2	☐ 1

© 2008-2016 PROMIS Health Organization and PROMIS Cooperative Group

		Without any difficulty	With a little difficulty	With some difficulty	With much difficulty	Unable to do
PF_65	How much difficulty do you currently have removing wrappings from small objects?	☐ 5	☐ 4	☐ 3	☐ 2	☐ 1
PF_7	Are you able to exercise regularly?	☐ 5	☐ 4	☐ 3	☐ 2	☐ 1
PF_9	Are you able to receive a call on a cell phone?	☐ 5	☐ 4	☐ 3	☐ 2	☐ 1
PFA12	Are you able to push open a heavy door?	☐ 5	☐ 4	☐ 3	☐ 2	☐ 1
PFA14	Are you able to carry a heavy object (over 10 pounds)?	☐ 5	☐ 4	☐ 3	☐ 2	☐ 1
PFA20	Are you able to cut your food using eating utensils?	☐ 5	☐ 4	☐ 3	☐ 2	☐ 1
PFA25	Are you able to do yard work like raking leaves, weeding, or pushing a lawn mower?	☐ 5	☐ 4	☐ 3	☐ 2	☐ 1
PFA31	Are you able to get up off the floor from lying on your back without help?	☐ 5	☐ 4	☐ 3	☐ 2	☐ 1
PFA33	Are you able to exercise hard for half an hour?	☐ 5	☐ 4	☐ 3	☐ 2	☐ 1
PFA36	Are you able to put on and take off a coat or jacket?	☐ 5	☐ 4	☐ 3	☐ 2	☐ 1
PFA38	Are you able to dry your back with a towel?	☐ 5	☐ 4	☐ 3	☐ 2	☐ 1
PFA43	Are you able to write with a pen or pencil?	☐ 5	☐ 4	☐ 3	☐ 2	☐ 1
PFA44	Are you able to put on a shirt or blouse?	☐ 5	☐ 4	☐ 3	☐ 2	☐ 1

© 2008-2016 PROMIS Health Organization and PROMIS Cooperative Group

		Without any difficulty	With a little difficulty	With some difficulty	With much difficulty	Unable to do
PFA45	Are you able to get out of bed into a chair?..	☐ 5	☐ 4	☐ 3	☐ 2	☐ 1
PFA50	Are you able to brush your teeth?.............	☐ 5	☐ 4	☐ 3	☐ 2	☐ 1
PFA51	Are you able to sit on the edge of a bed?...	☐ 5	☐ 4	☐ 3	☐ 2	☐ 1
PFA52	Are you able to tie your shoelaces?	☐ 5	☐ 4	☐ 3	☐ 2	☐ 1
PFA53	Are you able to run errands and shop?	☐ 5	☐ 4	☐ 3	☐ 2	☐ 1
PFA55	Are you able to wash and dry your body? .	☐ 5	☐ 4	☐ 3	☐ 2	☐ 1
PFA56	Are you able to get in and out of a car?.....	☐ 5	☐ 4	☐ 3	☐ 2	☐ 1
PFA8	Are you able to move a chair from one room to another?.......................................	☐ 5	☐ 4	☐ 3	☐ 2	☐ 1
PFA9	Are you able to bend down and pick up clothing from the floor?............................	☐ 5	☐ 4	☐ 3	☐ 2	☐ 1
PFB12	Are you able to make a bed, including spreading and tucking in bed sheets?.........	☐ 5	☐ 4	☐ 3	☐ 2	☐ 1
PFB15	Are you able to change the bulb in a table lamp?..	☐ 5	☐ 4	☐ 3	☐ 2	☐ 1
PFB20	Are you able to cut a piece of paper with scissors?	☐ 5	☐ 4	☐ 3	☐ 2	☐ 1
PFB21	Are you able to pick up coins from a table top?...	☐ 5	☐ 4	☐ 3	☐ 2	☐ 1
PFB22	Are you able to hold a plate full of food?..	☐ 5	☐ 4	☐ 3	☐ 2	☐ 1

© 2008-2016 PROMIS Health Organization and PROMIS Cooperative Group

		Without any difficulty	With a little difficulty	With some difficulty	With much difficulty	Unable to do
PFB26	Are you able to shampoo your hair?	☐ 5	☐ 4	☐ 3	☐ 2	☐ 1
PFB27	Are you able to tie a knot or a bow?	☐ 5	☐ 4	☐ 3	☐ 2	☐ 1
PFB34	Are you able to change a light bulb overhead?	☐ 5	☐ 4	☐ 3	☐ 2	☐ 1
PFB41	Are you able to trim your fingernails?	☐ 5	☐ 4	☐ 3	☐ 2	☐ 1
PFB8	Are you able to carry two bags filled with groceries 100 yards?	☐ 5	☐ 4	☐ 3	☐ 2	☐ 1
PFC42_PF	Are you able to open a tight or new jar?	☐ 5	☐ 4	☐ 3	☐ 2	☐ 1
PFC45	Are you able to get on and off the toilet? ..	☐ 5	☐ 4	☐ 3	☐ 2	☐ 1
PFC47	Are you able to be out of bed most of the day?	☐ 5	☐ 4	☐ 3	☐ 2	☐ 1
PFC49	Are you able to water a house plant?	☐ 5	☐ 4	☐ 3	☐ 2	☐ 1
PFC53	Are you able to get in and out of bed?	☐ 5	☐ 4	☐ 3	☐ 2	☐ 1
	**Group B Items (PF_16 thru PFC6)**	**Without any difficulty**	**With a little difficulty**	**With some difficulty**	**With much difficulty**	**Unable to do**
PF_16	Are you able to hike a couple of miles on uneven surfaces, including hills?	☐ 5	☐ 4	☐ 3	☐ 2	☐ 1
PF_17	Are you able to walk more than a mile?	☐ 5	☐ 4	☐ 3	☐ 2	☐ 1
PFA23	Are you able to go for a walk of at least 15 minutes?	☐ 5	☐ 4	☐ 3	☐ 2	☐ 1

© 2008-2016 PROMIS Health Organization and PROMIS Cooperative Group

	Group B Items (PF_16 thru PFC6)	Without any difficulty	With a little difficulty	With some difficulty	With much difficulty	Unable to do
PFA42	Are you able to carry a laundry basket up a flight of stairs?	☐ 5	☐ 4	☐ 3	☐ 2	☐ 1
PFB9	Are you able to jump up and down?	☐ 5	☐ 4	☐ 3	☐ 2	☐ 1
PFC13	Are you able to run 100 yards?	☐ 5	☐ 4	☐ 3	☐ 2	☐ 1
PFC32	Are you able to climb up 5 flights of stairs?	☐ 5	☐ 4	☐ 3	☐ 2	☐ 1
PFC38	Are you able to walk at a normal speed? ...	☐ 5	☐ 4	☐ 3	☐ 2	☐ 1
PFC6	Are you able to walk a block on flat ground?	☐ 5	☐ 4	☐ 3	☐ 2	☐ 1
	**Group C Items (PF_21 thru PFC40)**	**Without any difficulty**	**With a little difficulty**	**With some difficulty**	**With much difficulty**	**Unable to do**
PF_21	Are you able to go up and down stairs?	☐ 5	☐ 4	☐ 3	☐ 2	☐ 1
PF_3	Are you able to stand on a moving escalator?	☐ 5	☐ 4	☐ 3	☐ 2	☐ 1
PF_52	How much difficulty do you currently have standing up from a low, soft couch? .	☐ 5	☐ 4	☐ 3	☐ 2	☐ 1
PF_53	Are you able to stand upright briefly without support?	☐ 5	☐ 4	☐ 3	☐ 2	☐ 1
PFA10	Are you able to stand for one hour?	☐ 5	☐ 4	☐ 3	☐ 2	☐ 1
PFA37	Are you able to stand for short periods of time?	☐ 5	☐ 4	☐ 3	☐ 2	☐ 1
PFA41	Are you able to squat and get up?	☐ 5	☐ 4	☐ 3	☐ 2	☐ 1

© 2008-2016 PROMIS Health Organization and PROMIS Cooperative Group

	Group C Items (PF_21 thru PFC40)	Without any difficulty	With a little difficulty	With some difficulty	With much difficulty	Unable to do
PFB10	Are you able to climb up five steps?..........	☐ 5	☐ 4	☐ 3	☐ 2	☐ 1
PFB32	Are you able to stand unsupported for 10 minutes?...	☐ 5	☐ 4	☐ 3	☐ 2	☐ 1
PFC29	Are you able to walk up and down two steps?..	☐ 5	☐ 4	☐ 3	☐ 2	☐ 1
PFC40	Are you able to kneel on the floor?............	☐ 5	☐ 4	☐ 3	☐ 2	☐ 1

© 2008-2016 PROMIS Health Organization and PROMIS Cooperative Group
